# Perilous medicine in Tigray: a systematic review

**DOI:** 10.1186/s13031-023-00524-x

**Published:** 2023-05-30

**Authors:** Hailay Gesesew, Hafte Kebede, Kenfe Berhe, Nelsensius Fauk, Paul Ward

**Affiliations:** 1 College of Health Sciences, Mekelle University, Mekelle, Tigray Ethiopia; 2 Research Centre for Public Health, Equity and Human Flourishing, Torrens University Australia, Adelaide, SA Australia

**Keywords:** Attacks, Conflict, Health care, Perilous medicine, Systematic review, Tigray

## Abstract

**Background:**

The war in Tigray, North Ethiopia which started in November 2020, has destroyed decades of the region’s healthcare success. There is some emerging published evidence on attacks on health care in the region, and we synthesized the available evidence on ‘perilous medicine’ in Tigray to understand the data source, subjects and content covered, and what gaps exist.

**Methods:**

We employed a systematic review and performed a systematic search of MEDLINE, PubMed, CINHAL, Web of Science and Scopus. We included English written documents published from 4 November 2020 to 18–19 October 2022 and updated the search on 23 January 2023. HG and NF independently performed title, abstract and full-text screening. We used Joanna Briggs Institute (JBI) tools to appraise and extract data, and applied content synthesis to analyze. The PROSPERO registration number is CRD42022364964.

**Results:**

Our systematic review search yielded 8,039 documents, and we finally found 41 documents on conflict and health in Tigray. The areas were: (1) attacks on infrastructure, health or aid workers, patients, ambulances or aid trucks identified in 29 documents—the documents reported targeted attacks on health infrastructure and personnel; (2) interruption of health or social services in 31 documents—the documents reported medical and humanitarian siege; (3) outcomes and direct or indirect impacts in 33 documents—the documents reported increased magnitude of illnesses, and catastrophic humanitarian crises including the use of food, medicine and rape as tools of war; and (4) responses, rebuilding strategies, and recommendations in 21 documents—the documents reported improvisation of services, and calling to seize fire, accountability and allow humanitarian.

**Conclusions:**

Despite promising studies on conflict and health in Tigray, the documents lack quality of designs and data sources, and depth and diversity of subjects and contents covered; calling further primary studies on a prioritized future research agenda.

**Supplementary Information:**

The online version contains supplementary material available at 10.1186/s13031-023-00524-x.

## Introduction

Tigray, North Ethiopia, has faced a catastrophic armed conflict since 4 November 2020 [1]. The Ethiopian National Defence Forces, Amhara special forces and Amhara militia, and Foreign Eritrean army (hereafter referred to as allied forces unless mentioned individually) attacked Tigray from multiple fronts; and Tigray Defence Forces (TDF) were the defending forces on behalf of the Tigray Regional government and people [1]. Martin Plaut, in a book titled *The Tigray war & regional implications* (p.7) [2], described,

*This [Tigray] conflict began as what the Ethiopian Prime Minister, Abiy Ahmed, described as no more than a local “law enforcement operation” in November 2020. It soon escalated into a regional conflict involving Eritrean and Somali troops and Amhara special forces. The Tigrayans were rapidly driven from their capital, Mekelle and most of the region was soon in Ethiopian or Eritrean hands. Yet the Tigrayans fought on and in June 2021 transformed what had been a guerrilla war into a conventional conflict when they launched what they called “Operation Alula” ….*

The armed conflict in Tigray has violated declarations and resolutions [3] of the 1863 International Committee of the Red Cross (ICRC) and the 1864 international humanitarian law [4] leading to caused immense humanitarian crisis. Burki T [5], in their report to *The Lancet* (p. 1), stated,

*It is impossible to know exactly how bad things are in Tigray. Internet services have been shut down in the northern Ethiopian state since November, 2020. Telephone lines were cut in June, 2021; at the same time, the federal government in Addis Ababa imposed a punishing blockade. Barely any fuel has been permitted to enter, severely curtailing the prospects for travel, especially outside the state capital, Mekelle. The vast majority of the 7 million people who live in Tigray are struggling to find enough food. Almost half a million children are thought to be malnourished.*

In particular, there have been attacks on healthcare facilities and personnel (e.g. physical attacks, threats of attacks on health facilities and staff, attacks on ambulances and supply trucks), which have put the healthcare system of the region in a near totalcollapse [6, 7]. This is the focus of our paper—the impact of the war on healthcare facilities, services and personnel. From our systematic review, we identified gaps in the literature that can be used to identify required areas of future research on the impact of war on healthcare facilities, services and personnel.

**Fig. 1:**
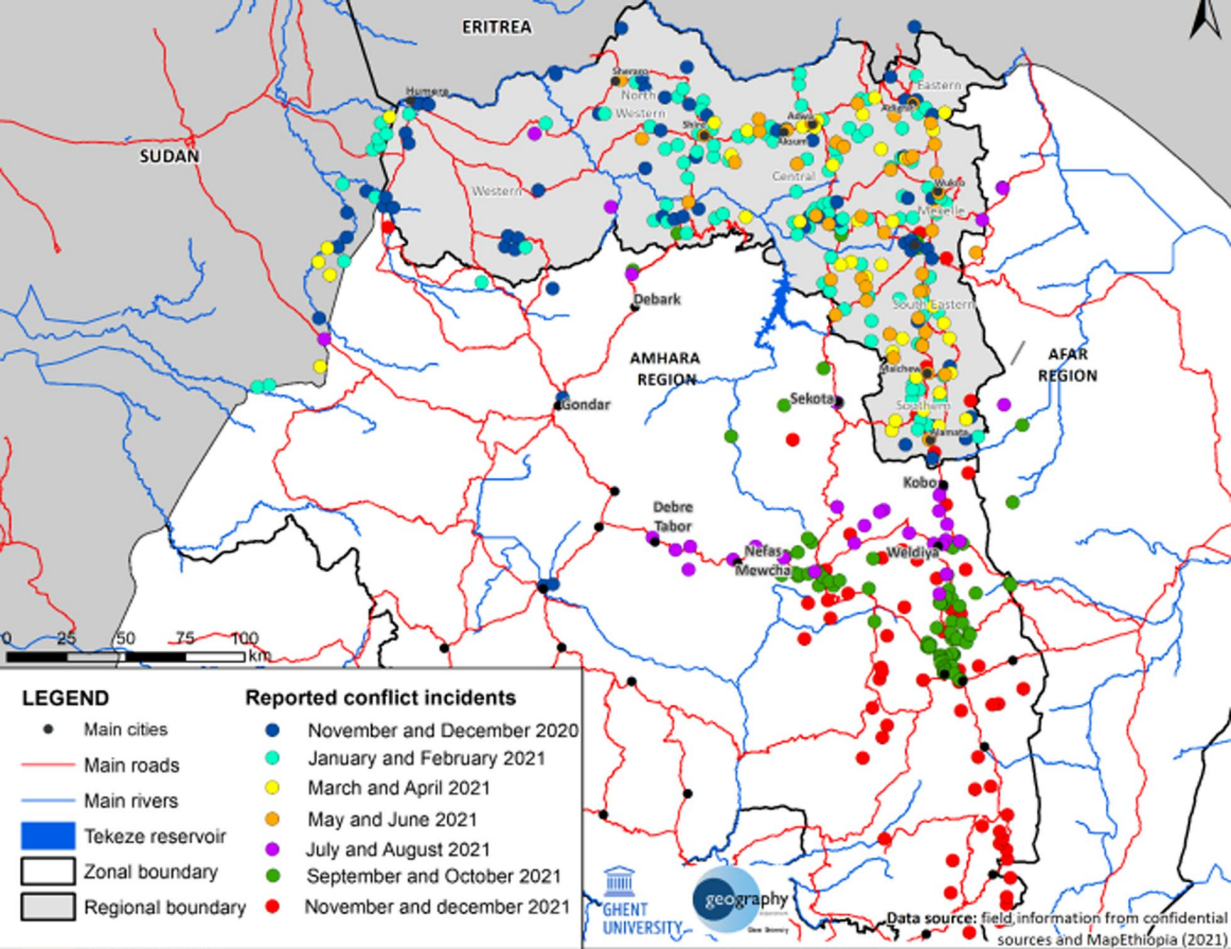
“Reported conflict incidents from November 2020 to December 2021. © Tigray: atlas of the humanitarian situation. Reported conflict incidents in the first fourteen months of the Tigray War, including battles, ambushes, air strikes, drone attacks and shelling (reported up to 21 December 2021).” (Ngussie and Hailu [8]). This figure describes the severity of limited access due to the conflict from November 2020 to December 2021

The Federal government of Ethiopia has also imposed a siege on and blocked aid to Tigray, such as medicine, food and fuels, and shut down its essential services, such as electricity, banking, internet and telecommunications [5–7]. Figure 1 describes the reported conflict incidents in Tigray from November 2020 to December 2021 [8]. This has caused immense causalities and dire humanitarian situations. Despite the scale of the atrocities and for a more extended period (for almost two years), the armed conflict in Tigray is described as being among the most neglected and less documented contemporary conflicts, which unfortunately was concealed by the global attention to the Russian-Ukraine war [9].

A handful of international organizations, including Médecins Sans Frontières (MSF), Norway Refugee Council (NRC), and United Nations Office for the Coordination of Humanitarian Affairs (UNOCHA), received the permission to operate in limited areas, under constant pressure and with limited access to internet and telecommunication services in the region. Henceforth, new reports, viewpoints, editorials, correspondence, and lately peer reviewed articles started to be published on the impact of the war on health services and health systems. The primary author of this review, along with other colleagues, has published the first peer reviewed paper [7] and a detailed viewpoint [10] on the impact of the armed conflict on the entire healthcare system of the region. Nevertheless, the available research outputs were not synthesized (as per our initial search) to identify gaps and guide a future research agenda. The present study aims to synthesize the available evidence through a systematic review, which can hopefully be used to develop a future research agenda.

Undertaking a systematic review and developing a research agenda on the impact of the war in Tigray is crucial for several reasons. First, understanding the scale, scope and impact of the attacks on the healthcare system helps the Tigray government and global humanitarian actors to allocate resources and identify targeted programs during the crisis and recovery process, as peace agreement between Ethiopia’s federal government and Tigray’s regional government [11] was signed on 2 November 2022. The promising peace agreement contains ceasefire, lifting the imposed siege, opening humanitarian corridors, and resuming social services in Tigray. Second, better documentation helps to understand the true scope of the attacks on the healthcare system of the region to assist advocacy mechanisms and accountability for the perpetrators. Third, Tigray in general is looking forward to rebuilding damaged infrastructure and replacing the human health drain and at least its status quo ante. Fourth, evidence of future research findings is indispensable.

## Methods

### Systematic review search strategy and selection criteria

We applied a systematic review using evidence from quantitative and qualitative studies or reports (documents hereafter). We followed the Preferred Reporting Items for Systematic Reviews and Meta-Analyses (PRISMA) guidelines for the systematic review (Additional file 1: Table S1) [12]. We considered English written peer-reviewed articles and reports from governmental and non-governmental reports between November 2020 and October 2022. We included original articles, editorials, commentaries and correspondences published in English in peer reviewed journals listed in Scimago Journal Rankings (*SJR*); and reports of international organizations on armed conflict and health in Tigray. We excluded opinion pieces or viewpoints published in media outlets. We also excluded reports focusing on broad humanitarian perspectives without describing the cause of the humanitarian crisis due to conflict.

We conducted a systematic search on MEDLINE, PubMed, CINHAL, Web of Science and Scopus for English written documents. The timelines of documents are limited between 4 November 2020 (the start of the conflict) and 18–19 October 2022 (the date of the systematic search), but alerts in the databases were also turned on to receive additional documents, and we finally updated the search on 23 January 2023. We developed words and synonyms for three keywords for the search: conflict, health and Tigray. A comprehensive search strategy for each database is presented in Additional file 1: Table S2. We then ran the systematic search strategy in each database, exported to Endnote and removed duplicates, exported to Covidence, and then screened the titles and abstracts in the Covidence system. We also searched for grey literature in selected sources from the *Grey Literature in Health Research* [13] using keywords ‘conflict AND health AND Tigray’. We contacted MSF, UNOCHA, and NRC as they have been actively delivering health services and other humanitarian aids in Tigray during the crisis period. A targeted search was also conducted to the following sources: MSF, UNOCHA, and BMC Conflict and Health Journal using the following concepts (and their keywords): Conflict AND Health AND Tigray. Bibliographies of all shortlisted articles were reviewed to identify additional relevant studies.

Within the search strategy, we define attacks as violence, threatened/actual intimidation and interference, and misuse or misrepresentation of the protected status of healthcare, such as the use of health facilities for treating troops and denying civilians. We define health services as; facilities and buildings, transport routes or vehicles used for patients, medicines or other health technologies, patients, and healthcare personnel. Conflict is defined based on the international humanitarian law (IHL) definitions of international armed conflicts and non-international armed conflicts. However, we will remain open to additional definitions by authors of identified literature. For the Delphi method, experts with any form of conflict of interest will be excluded.

We conducted quality appraisals for the commentaries, original articles and case studies using the JBI checklists (Additional file 1: Tables S3a-3c), but included and extracted data from all relevant documents irrespective of their quality score. Two independent reviewers (HAG and NKF) performed screening for the title and abstract, and both reviewers independently and blindly labelled each study with reasons for inclusion and exclusion. HAG and NKF then independently screened the full-text using the *Joanna Briggs Institute* (*JBI*) appraisal instrument. Discussions among all research team members were carried out on ambiguities, conflicts, and further exclusion decisions. A flow chart is used to map the number of documents retrieved from databases, screened papers, eligible papers, and included studies.

### Data extraction and analysis

To extract data, we used the JBI standardized data extraction instrument as a guide. We piloted the data extraction tool, employed a thorough discussion with the team members (HAG, NKF and PW developed initial themes), uploaded the refined extraction checklist to the Covidence system, and finally extracted data from the included studies. HAG and NKF were involved in the data extraction. The extracted data were read and discussed in detail by all team members before employing the final development of themes and subthemes. The characteristics of the studies were described and the available documents were compiled and summarized with frequency and bar graphs. The findings from the selected studies were thematized using content analysis [14].

## Results

### Characteristics of studies

**Fig. 2:**
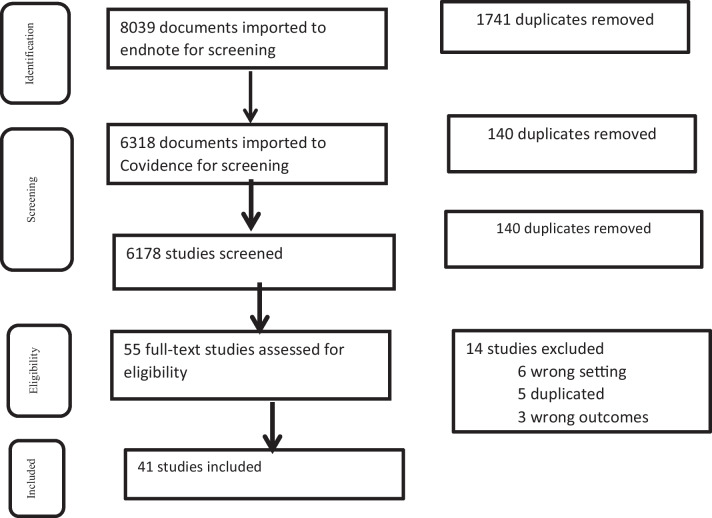
Flow chart of study selection conflict and health in Tigray, 2020–2022. This figure describes the pictorial schematic presentation/ flow chart of studies included in the systematic review

**Fig. 3:**
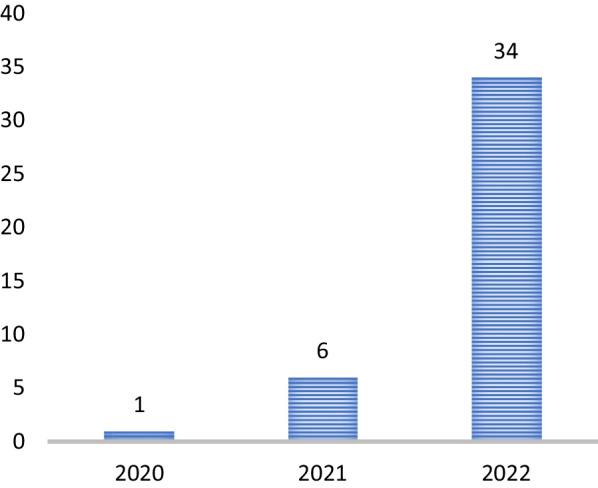
Number of records on perilous medicine in Tigray published between November 2020 and October 2022 (n = 41). This figure describes the number of documents on conflict and health in Tigray

**Fig. 4:**
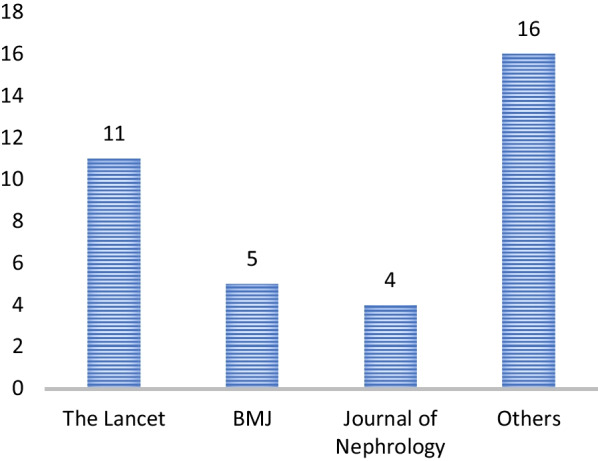
Journals where articles, commentaries, and case studies are published in (n = 36). This figure describes the list of journals where the included studies are published

Our systematic review search yielded 8,039 documents. In total, 33 records were screened for full text review, and additional 17 records were found through bibliographical and manual checking (we have received two letters and a report from the Tigray government). The updated search was done on 23 January 2023 and database alerts provided five additional relevant documents. We contacted MSF, UNOCHA, and NRC on 24/10/2022 if there was any report on conflict and health in Tigray between 2020 and 2022, but none of them responded until the submission date of the paper for publication. Finally, we identified 41 documents for data extraction [5–7, 15–52] (Fig. 2). Of all documents, 24 are commentaries [5, 6, 15–36], 9 are original articles [7, 37–44] (2 studies used primary data), 3 are reports [45–47], 3 are letters [48–50], and 2 are case studies [51, 52]; and 34 studies were published in 2022 (Fig. 3). About half of the articles, commentaries, and case studies were published in *The Lancet* and BMJ families whereas the other half were published in Nature Medicine, JAMA Oncology, BMC Medicine, and others (Fig. 4).

### Focus of thematic areas

The majority of included documents focused on targeted attacks on health facilities and ambulances, followed by lack of or collapse of the entire health care service. Below, we present the review findings by describing the number of documents which mentioned the themes and subthemes from Table 1, key findings of the themes and subthemes from Additional file 1: Table S4a, and illustrative examples of the key findings from Additional file 1: Table S4b.

**Table 1:** Number of documents identifying key issues on conflict & health in Tigray, North Ethiopia, 2020–2022

Key themes and subthemes	n
Attacks on facilities and personnel [5–7, 15, 16, 18–22, 24–33, 35, 36, 38, 39, 44–46, 49, 52]	29
Infrastructure [7, 15, 16, 18–22, 24–26, 28–32, 35, 36, 38, 39, 44–46, 49]	24
Health centre [7, 20, 21, 24–26, 28, 29, 31, 39, 44–46]	13
Hospital [7, 16, 19, 21, 24–26, 28, 39, 44, 46]	11
Health post [7, 21, 25, 26, 39, 44, 46]	7
Personnel/aid workers [5–7, 19–21, 24, 27, 31, 44–46, 52]	13
Aid workers [5, 31, 45, 46, 52]	5
Health workers in Tigray health facilities [5–7, 19–21, 24, 27, 44–46, 52]	12
No salary payment [5–7, 27, 46]	5
Killed [20, 24, 46, 52]	4
Displacement [7, 19, 44, 46]	4
Threatened, harassed, or blocked not deliver services [21, 44–46]	4
Ambulance/aid trucks [5, 7, 19, 20, 24, 32, 33, 45, 46]	9
Ambulance [5, 7, 19, 20, 24, 46]	6
Aid trucks/NGO vehicles [32, 33, 45, 46]	4
Closing roads/checkpoints to patients [18, 30, 35, 45, 46]	5
Interruptions/lack of social or health services [5–7, 15–29, 31–33, 35, 36, 41, 44–46, 48–50, 52]	31
Siege and interruption of social services [5, 6, 17, 19, 20, 23, 25–29, 44–46, 48]	15
Siege [5, 6, 17, 25, 26, 28, 29, 46]	8
Telecommunications [5, 17, 20, 25, 29, 46, 48]	7
Fuels [17, 25, 29, 44, 46, 48]	6
Banking [5, 17, 23, 25, 46, 48]	6
Suspension of NGOs (MSF and NRC) [5, 27, 31, 46]	4
Interruption of health services [5–7, 15–22, 24–28, 31–33, 35, 36, 41, 45, 46, 48–50, 52]	28
Shortage or lack of basic/emergency diagnostic supplies and medications (e.g. shortage of glove, insulin, or diagnostic tests, or cancelation of surgery) [5–7, 15, 20, 22, 24, 25, 27, 28, 31, 33, 35, 45, 46, 48, 49, 52]	18
Interruption of NCDs services [5–7, 15–18, 21, 22, 25–27, 32, 35, 36, 41, 45, 46, 50]	19
HIV [5, 27, 32, 41, 45, 46]	6
Cancer services [17, 22, 25, 35, 46]	5
Renal diseases [6, 15, 16, 46]	4
Diabetic mellitus [32, 46, 50]	3
Cardiac illnesses [32, 36]	2
Tuberculosis [5, 46]	2
Stoppage/interruption of child vaccination services [5, 31, 45, 46, 49]	5
Collapse of maternal health services [7, 26, 46]	3
Stoppage of health extension program [7, 19, 46]	3
Interruption of Covid-19 services [5, 20, 46]	3
Outcomes/impacts resulted from attacks/interruptions [5, 7, 15–21, 23, 24, 26–38, 40–42, 44–49]	33
Food insecurity [7, 19, 20, 23, 28–30, 34, 35, 42, 46, 47]	12
Malnutrition [5, 7, 20, 21, 23, 26, 29, 31, 34, 46, 47]	11
Children [5, 7, 20, 23, 29, 31, 34, 46, 47]	9
Pregnant and lactating mothers [5, 7, 20, 26, 29, 31, 46, 47]	8
Doctors [21, 23]	2
Displacement (external [21, 28, 33, 46]) [5, 7, 20, 21, 28, 30, 33, 35, 36, 46, 48]	11 (4)
Deaths (Massacre [5, 21, 24, 28, 32, 45]) [5, 21, 24, 27, 28, 32, 35, 36, 45, 46, 48]	11 (6)
Sexual & gender-based violence [5, 7, 19–21, 24, 26, 28, 32, 35, 44–46]	17
Reported lost-to-follow-up or mortality of NCDs patients [7, 15, 16, 20, 32, 37, 40, 41, 46]	9
HIV [20, 32, 41, 46]	4
Diabetic mellitus [7, 32, 46]	3
Renal diseases [15, 16, 37]	3
Cardiac illnesses [32, 40]	2
Cancer patients [48]	1
Tuberculosis [46]	1
Outbreaks [5, 7, 29–31, 46, 49]	7
Malaria [5, 7, 30, 46]	3
Vaccine preventable illnesses [7, 46, 49]	3
Cholera [5, 7, 46]	3
Fistula [18, 26]	2
Mental health [38]	1
Vicarious trauma to health workers [15–17, 27]	4
Violations (ethnic cleansing [5, 32]) [5, 28, 32, 38, 46]	5 (2)
Response, rebuilding, and recommendations [6, 15, 16, 18, 20, 21, 23, 24, 28, 29, 35–37, 39, 43–45, 48, 50–52]	21
Response such as using expired drugs or chemotherapies, re-washing gloves [15, 29, 48, 51, 52]	5
Rebuilding such as considering health extension program or training health workers [18, 20, 24, 43–45]	6
Suggestions/recommendations [6, 15, 16, 20, 21, 23, 28, 35–37, 39, 45, 48, 50, 51]	15
Call to lift siege, ceasefire, or allow to humanitarian aid [16, 20, 21, 23, 28, 35–37, 45, 48]	10
Call to professional associations to advocate or support medical aids [15, 28, 36, 48, 50, 51]	6
Accountability [19, 26, 45, 46]	4
Call to solidarity of health workers [6, 20, 23]	3
Limitation/methodological challenge [7, 47]	2

#### Attacks on health infrastructure and personnel

The majority of the documents (29) described attacks on health infrastructures, i.e. hospitals, health centres and health posts, health or aid workers, patients, ambulances or aid trucks. Twenty-four (24) documents described attacks on infrastructure [7, 15, 16, 18–22, 24–26, 28–32, 35, 36, 38, 39, 44–46, 49], 13 on health personnel or aid workers [5–7, 19–21, 24, 27, 31, 44–46, 52], 9 on ambulances or aid trucks [5, 7, 19, 20, 24, 32, 33, 45, 46], and 5 reported closing roads/checkpoints to patients or gender-based survivors [18, 30, 35, 45, 46]. The studies found that health facilities were deliberately destroyed, vandalized, occupied, and/or used as weapon of war. For example, Cousins S [18] reported the destruction of more than 80% of health facilities, including attacks on hospitals by drones and jets, as also reported by Hadera et al. [27]. Abebe et al. [44] reported militants occupied health facilities and Tigray Regional Health Bureau (TRHB) [46] reported some health facilities served as military camps for the Ethiopian and Eritrean troops for several months. Ambulances were also looted and damaged, and aid trucks were attacked. For example, Burki [5] reported that the number of ambulances that served the healthcare system in the region had been reduced from 312 to 38. Devi [33] reported blockade of aid trucks with humanitarian aids.

Healthcare professionals working in health facilities were also attacked, i.e. harassed, denied their salary, blocked from delivering services to civilians, displaced and even killed. For example, Clarfield et al. [6] and Burki [5] reported the denial of savings and salary of health workers by the Ethiopian Federal government for over a year, where such salary and savings denial led even to the malnutrition of doctors as reported by Paltiel et al. [23]. TRHB [46] reported the death of 37 and the displacement of many health workers as was also reported by Gebregziabher et al. [19] where more than 50% of members of the regional workforce were unable to report to their working institutions. Aid workers of different NGOs were also threatened. For example, *The Lancet*’s medical reporter, Devi S, reported that UN aid workers were threatened by airstrike [31] and stopped by an Ethiopian military convoy even if they had MSF-marked vehicle [32]; Human Rights Watch (HRW) [45] revealed the intimidation of health workers and humanitarian aid providers in Tigray, and Gebrearegay et al. [52] reported the killing of 22 humanitarian aid workers.

Regarding road blocks (or blocks at check points), Devi [30] described how the displacement of around 1.7 million people in northern Ethiopia and road blocks and military restrictions devasted the residents; Teka et al. [35] reported the blockade of civilian patients at checkpoints and near health facilities from accessing health services; and HRW [45] specifically reported the blockade of rape survivors at checkpoints while they attempted to seek care.

#### Interruption or lack of health or social services

This theme describes the interruption or blockade of health and social services as a result of attacks on health infrastructure and personnel. Fifteen documents reported medical or humanitarian siege or the interruption of social services [5, 6, 17, 19, 20, 23, 25–29, 44–46, 48] such as fuels [17, 25, 29, 44, 46, 48], telecommunications [5, 17, 20, 25, 29, 46, 48], and banking [5, 17, 23, 25, 46, 48] services. Devi [29] reported that Tigray were under systematic blockade of telecommunication, fuel, cash, food, and medications; and Teka et al. [25] and Hiluf et al. [17] reported total humanitarian and medical siege, respectively.

About 70% of the documents (28) reported the interruption of health services in Tigray [5–7, 15–22, 24–28, 31–33, 35, 36, 41, 45, 46, 48–50, 52]. For example, Makoni [22] described that none of the health facilities in the region operated at their pre-war level. Similar findings were reported by Tesema et al. [24] suggesting that the three tier health structure at the pre-war period, i.e. primary, secondary, and tertiary care and the linkage among the tiers was non-existent during the war period. This is supported by Gesesew et al. [7] who reported that the structure of the district (locally known as *Woreda*) Health Office was non-functional. Of the 28 documents, 18 reported the collapse of Tigray healthcare system reflected in the shortage or lack of basic or emergency diagnostic supplies and medications such as shortage of gloves, insulin, diagnostic tests, or cancellation of surgery [5–7, 15, 20, 22, 24, 25, 27, 28, 31, 33, 35, 45, 46, 48, 49, 52]. For instance, Hadera et al. [27] reported the cancellation of more than 3700 surgeries due to lack of oxygen in 12 months; Teka et al. [25] revealed a lack of oxygen, anaesthetic medications, antibiotics, and operation theatre materials; and Devi S [33] reported a lack of sutures, antibiotics, painkillers and gloves where single-use items such as gloves, surgical materials, and even chest drains were washed and reused; and in some places, doctors have replaced disinfectant with salt to clean wounds. NGOs (MSF and NRC) delivering mobile health services were also suspended [5, 27, 31, 46].

Interruption of services to chronic communicable and non-communicable diseases (NCDs) was reported by 19 documents [5–7, 15–18, 21, 22, 25–27, 32, 35, 36, 41, 45, 46, 50]. This included the stoppage of dialysis service in Ayder Referral Hospital [15], lack of basic diabetic care [17], and the closure of cancer clinic in Ayder [22]. Additional documents showed a sharp decline in the number of follow ups of hypertension patients by 85%, 59%, 85%, 100%, and 11% in Eastern, North-western, South eastern, and Southern zones, respectively [40]; and a dramatic decline of Cath Lab procedures leading to decreasing cardiac patients followed in the cardiology unit by 50% [36]. Weledegebriel et al. [41] also reported a sharp decline in HIV services, clinical follow-ups, laboratory services, and ART.

Vaccination services, one of the key primary health care services in Tigray, were reported as collapsed by 5 documents [5, 31, 45, 46, 49]. Burki [5] reported childhood vaccination rates declined from 73% before the war to 27% during the war, as also supported by Devi [31] who reported missing of critical vaccinations by nearly 200,000 children. The coverage of the first dose of the measles vaccine has dropped from 83 to 28%, whereas nearly 900,000 children younger than 5 years have missed the polio vaccine [5]. Some other documents reported on the collapse of maternal health services [7, 26, 46], stoppage of health extension program [7, 19, 46], and interruption of Covid-19 care services [5, 20, 46]. Gesesew et al. [7] revealed that maternal and child health services (including vaccination) were collapsed. For example, antenatal care and skilled delivery decreased from 94 to 16% and 81% to 21% in 2019 to 2022, respectively [7].

#### Outcomes or impacts

This theme describes the outcomes and direct or indirect impacts of the interruption or blockade of health and social services resulting from the attacks on infrastructures, health or aid workers, patients, ambulances or aid trucks as presented in the previous themes. In this theme, 12 documents reported about food insecurity, and some of them mentioned the weaponization of food [7, 19, 20, 23, 28–30, 34, 35, 42, 46, 47]. In their report to *The Lancet*, Burki [5] reported the struggle of the vast majority of the 7 million people who live in Tigray to find enough food and Devi [30] stated that 92% of the population in the region faced acute food insecurity in 2021.

Malnutrition was assessed by 11 documents [5, 7, 20, 21, 23, 26, 29, 31, 34, 46, 47]. Burki [5] reported that half a million children were thought to be malnourished, and Devi [29] corroborated this by reporting that 13% of children younger than 5 years and half of pregnant and breastfeeding women were malnourished in Tigray. Similarly, World Food Program (WFP) [47] and TRHB [46] revealed increased malnutrition among children, and pregnant and lactating mothers. Wall [21] even reported malnutrition of doctors, and Paltiel et al. 2022 [23] reported hunger in health workers to the extent that nurses fainted in the hospital while supporting patients.

Displacement was reported by 11 documents [5, 7, 20, 21, 28, 30, 33, 35, 36, 46, 48] where 4 documents reported displacement to Sudan [21, 28, 33, 46]. For example, TRHB [46] reported that 1.2 Million Tigrayans in Western Tigray had been forcibly evicted from their homes, currently in a destitute life as IDPs within Tigray, while over 70,000 people fled to Sudan. Similarly, the report by Yemane et al. [28] suggested that two million Tigrayans have become displaced internally, many fleeing to Mekelle, and Some 70,000 Tigrayans have fled across the western border to Sudan as refugees. This is also supported by Wall [21] reporting that thousands of Tigrayans fled across the western border, seeking refuge in Sudan. Therefore, Mulugeta et al. [34] concluded that a total blockade of humanitarian aid, displacement of millions of people, and wanton destruction of health facilities are some of the defining characteristics of Tigray’s armed conflict. Eleven documents reported civilian deaths [5, 7, 20, 21, 28, 30, 33, 35, 36, 46, 48] of which 6 documents reported massacres [5, 21, 24, 28, 32, 45]. For example, Devi S [32] reported that almost 2000 people were killed in more than 150 massacres by soldiers. Wall [21] reported that thousands of civilians were killed, often in extrajudicial executions.

Conflict related gender-based violence was reported by 17 documents, including the use of rape as a tool of war [5, 7, 19–21, 24, 26, 28, 32, 35, 44–46]. For example, Devi [32] reported that armed actors raped women and girls in front of family members, and men were forced to rape their own family members under the threat of violence. It is also reported that Ethiopian and Eritrean soldiers have used gang rape and the intentional spread of HIV to the women and girls of Tigray as weapons of war [20]. This is also supported in the report by HRW [45] emphasizing the weaponization of rape, where the age of the victims ranged between 6 and 80 years.

In relation to the massacre, and weaponization of food, medication and rape, 5 documents reported human right violations [5, 28, 32, 38, 46]. For example, a document by Burki [5] reported forced displacement, sexual violence, and mass killings, as well as serious violations of international humanitarian law. TRHB [46] claimed the violation of the Geneva Convention as the entire seven million Tigrayans were under a total communication blackout, life threatening air and drone strikes, and suffering from a lack of basic life support amenities, such as food, shelter, water, medicines, cash, and fuel for over a year. Two documents by Burki [5] and Devi [32] mentioned ethnic cleansing in Western Tigray.

Two documents, Cousins [18] and Gesesew et al. [26], reported about the insurgence of fistula and its psychological impact on women and girls; and one document, Favara et al. [38], assessed mental illness among youths and found that rates of at least mild anxiety were eleven times higher and mild depression more than doubled than pre-conflict. Seven documents reported outbreaks of different illnesses, including malaria and vaccine preventable illnesses [5, 7, 29–31, 46, 49]. For example, Devi [30] reported the expected rise of malaria outbreaks; and TRHB [49] reported outbreaks of vaccine preventable illnesses, including measles and polio. There are 9 documents that reported lost-to-follow-up or mortality of NCDs patients [7, 15, 16, 20, 32, 37, 40, 41, 46]. For example, TRHB [46] reported 90% loss of-to-follow-up rate of tuberculosis patients. Berhe et al. [15] also reported the increment of mortality rate from 25.5% (28 of 110 patients) in 2020 to 53.1% (43 of 81 patients) in 2021 in patients receiving hemodialysis in Ayder Referral Hospital resulting from the stoppage of dialysis service.

Vicarious trauma in health workers by 4 documents [15–17, 27]. For example, Berhe et al. [15] revealed that the painful deaths of veterans and new patients had imposed huge psychological burdens on the staff. Hiluf et al. [17] also showed that health workers cried when they lost patients on their hands due to a lack of medications and diagnostic supplies.

#### Responses, rebuilding, or recommendations

This theme describes about interventions or responses employed, potential rebuilding strategies reported, and suggestions or recommendations identified in the documents. At least four documents reported about the responses to the ongoing healthcare crisis. Given the near-total collapse of the healthcare system, 5 documents reported what the responses to the health crisis were, such as using expired drugs or chemotherapies or re-washing gloves [15, 29, 48, 51, 52]; and 6 documents reported about how to rebuild the collapsed healthcare system such as considering health extension program or training health workers [18, 20, 24, 43–45].

Whilst 10 documents reported regarding the call for action to lift the siege, ceasefire, or allowance of humanitarian aid [16, 20, 21, 23, 28, 35–37, 45, 48], 6 documents specifically requested targeted professional associations (e.g. oncologist, nephrologist, etc.) to show their solidarity to patients or the health workers losing patients at hand as a result of medical besieging [15, 28, 36, 48, 50, 51]. For example, Weldegerima et al. [36] called cardiology communities to advocate allowance of medical supplies to cardiac and other patients in Tigray from the Ethiopian government. Whilst 5 documents reported on violations to international declarations or resolutions, 4 documents reported on ensuring accountability to perpetrators.

## Discussion

To the best of our knowledge, this is the first study to synthesize the available evidence on the perilous medicine in Tigray. Whilst 36 studies were published in high-impact journals such as *The Lancet*, only two studies used primary data. There are at least 31 studies describing service interruptions to NCDs or associated outcomes such as increased incidence, prevalence, lost-to-follow-up, other complications and mortality, and only one study covered mental illnesses. However, the majority of these studies were conducted in a limited population (adults) and setting (Ayder referral Hospital). Western and Southern Tigray were not included in almost all studies except in humanitarian crises such as displacement, massacre, and ethnic cleansing. Excluding the three reports, the assessment of perilous medicine stratified by zones in the Tigray region was also limited to one study each to topics on assessing damage to health infrastructure, HIV care outcomes, and hypertension follow up. This evidence shows wider gaps in unaddressed health issues among different populations calling for the employment of extensive studies.

While some studies discussed about the attack to (13) or vicarious trauma (4) among health/aid workers, only 3 studies reported solidarity to health workers. Similarly, there are reports on the deliberate destruction of health facilities, weaponization of food, medication, and rape, and even documents on violations of international laws, including ethnic cleansing. Nevertheless, only 4 studies discussed issues related to accountability and one study suggested that conflict should be a public health priority. Studies should be employed to investigate the prosecution of persons responsible for the most serious crimes, and name perpetrators and incorporate an accountability mechanism. In his *Perilous Medicine* book, Professor Leonard Rubenstein reported the loss of 30 million civilians in armed conflicts in the past three decades globally and called for conflict to be a serious public health agenda [53].

Our review found 29 documents that reported on attacks on health infrastructure or personnel, but the attacks were not characterized in detail using WHO’s Surveillance System for Attacks on Health Care (SSA) [54] in all zones in Tigray. It is supposed that the SSA systematically gather data on the attacks on health care to facilitate accountability and safeguard health and human rights. Yet, the WHO leadership did not perform the surveillance data on attacks on health care in Tigray—the database shows ‘0’ to Ethiopia while there are outputs in other conflict areas, such as Ukraine, Syria, Yemen, Afghanistan and other countries [55]. This may be due to several reasons. For example, WHO has repeatedly described that the Ethiopian government blocked access to Tigray. For example, Director General Dr Tedros *Adhanom Ghebreyesus* said, “… *cannot send supplies and medicines to Tigray because it’s under blockade, and the blockade is systematic*” [56]. Such geographical limitations and political restrictions by the Ethiopian government or failure of the incompetence of UN/WHO agencies in fulfilling their mandate on the issue undermines efforts of engagement with perpetrators who attacked health facilities and weakens prevention of future attacks [57].

The conflict was stopped in November 2022, and there are promising steps in the implementation of the signed peace agreement [11] opens opportunities to explore the myriad miseries in the collapsed health care system in Tigray, develop prioritized research agenda, and suggest prioritized action items for local, regional, federal, and global actors to inform short-, medium-, and long-term goals to rebuild the collapsed health care system in Tigray. The conflict in Tigray has caused one of the largest global humanitarian crises and the worst ever in Tigray's history. Paleoanthropologist and Nobel prize winner Professor Zeresenay Alemseged and other Tigrayan scholars [58] estimated more than 600,000 people (nearly 10% of Tigray’s population) have died [59], about one-third of its pre-conflict population remain displaced [30], and 70–80% of its health facilities were vandalized or damaged [7, 18] along with multidimensional health effects [7, 18, 30].

In light of the scale of the crisis, the urgency of the Tigray question and the need for advocacy on priority issues, a collaborative networking between institutions in Tigray and experienced institutions in conflict and health studies globally could be beneficial to conduct high level contextual studies. For example, *The Lancet* and the American University of Beirut organized a commissioned study on Syria conflict and health [60], and produced: (a) Comments, Perspectives and Correspondence; (b) research including reviews, and health policy using primary studies; and (c) organized policy discussions at important policy or political meetings such as the 72nd UN General Assembly and produced relevant material, and developed/developing several knowledge resources on different topics related to health and the Syrian conflict/war. This kind of crucial collaborative project could be replicated in Tigray with some of its institutions, such as Mekelle University and Tigray Health Research Institute. Such collaborative act may investigate the cost of war on the lives of Tigrayans, the destruction of Tigray’s health services, the failure of the global community and humanitarian system to respond effectively, the tragedy for Tigrayan refugees, the impact of the crisis on Tigray’s long-term social and economic development, current priority actions, and future scenarios.

We found no original article focusing on the rebuilding of the healthcare system of Tigray. We call that prioritized research agenda should also be identified using standard consensus studies, such as Delphi Technique [61] or other approaches. No document assessed the impact on the population and community health using population- or community-based surveys. This implies: (a) more large-scale primary studies are needed to know the magnitude and severity of conflict-caused health problems in Tigray; (b) when there is communication blackout, such as in the case of Tigray, data collection may be required to be assisted using technologies, such as satellite images [62], artificial inetllegence [63], and machine learning [64]; and (c) new methodologies are needed to characterize the attacks, estimate the extent of damage during the conflict, and project the short-, medium-, and long-term complications of the crisis.

While this study is the first to produce synthesized evidence on perilous medicine in Tigray, it has the following limitations. First, it excludes medical research on the impact of armed conflict on animals and plants, which affects the health of humans, implying the need for further research using the One-Health Approach. However, we tried to include zoonotic illnesses. Second, despite 4 documents mentioning the displacement of 60,000–70,000 Tigrayans to Sudan, our analysis did not assess studies on the health of Tigrayan refugees as the setting was limited by Tigray. However, we conducted a quick and dirty search and got no document. Third, acknowledging the positionality of the authors in the included studies, some of them are members of the Tigray community working to advocate against attacks on the region’s healthcare system. We believe this positionality informs the depth and range of collective experience to bring greater clarity and nuance to this paper.

## Conclusions

Overall, there are promising studies on perilous medicine in Tigray. The findings from this study are timely and critically important for several reasons for policy makers, humanitarian advocates, and researchers to benefit the people of Tigray and other populations in conflict settings. First, understanding the scale, scope and impact of the attacks on the healthcare system helps the Tigray government and global humanitarian actors allocate resources and identify targeted programs during recovery process. Second, better documentation helps to understand the true scope of the attacks on the healthcare system of the region so as to assist advocacy mechanisms and accountability for the perpetrators. Third, Tigray in general is looking forward to rebuilding damaged infrastructure, and replacing the human health drain, and at least its status quo ante and evidence of prioritized research findings are indispensable.

However, the documents are nowhere to the scale of the crisis, lack quality in designs and data sources, and are limited in depth and diversity of subjects and contents covered, indicating further research on a prioritized agenda. Comprehensive and collaborative research is needed on the health of people inside Tigray; the health of Tigrayan refugees; pillars of Tigray healthcare systems, including health workers, delivery, infrastructure, and transition to rebuilding; challenges to the international response to the crisis and humanitarian blockade; accountability to health-related international law violations; and policy options and next steps. The promising steps in the implementation of the signed peace agreement [11] open opportunities for such investigation and implementation of its outputs.

## Supplementary Information

**Additional file 1.** The additional file reports the PRISMA Checklist of items to include when reporting a systematic review or metaanalysis.

## Data Availability

All data relevant to the study are included in the article or uploaded as supplementary information. All data relevant to the study are included in the article.

## Abbreviations

ICRC: International Committee of the Red Cross
MSF: Médecins Sans Frontières
NRC: Norway Refugee Council
PRISMA: Preferred Reporting Items for Systematic Reviews and Meta-Analyses (PRISMA)
TDF: Tigray Defence Forces
UNOCHA: United Nations Office for the Coordination of Humanitarian Affairs
