# Supplementary material for: Perilous medicine in Tigray: a systematic review
Source: Confl Health. 2023 May 30;17:26. doi: 10.1186/s13031-023-00524-x (PMC10228460; doi:10.1186/s13031-023-00524-x)
Supplement: Supplementary file 1 — Additional file 1. The additional file reports the PRISMA Checklist of items to include when reporting a systematic review or metaanalysis. [file 13031_2023_524_MOESM1_ESM.pdf]

Perilious Medicine in Tigray: A systematic Review

Supplementary Tables

Supplementary table 1: PRISMA Checklist of items to include when reporting a systematic review or meta-analysis [Adapted from *Preferred reporting items for systematic review and meta-analysis protocols (PRISMA-P) 2015 statement* **by Moher D et al, 2015**]

Section/topic	#	Checklist item	Information rted Yes	Page ber(s) No	
**ADMINISTRATIVE RMATION**					
**Title**					
Identification	1a	Identify the report as a protocol of a systematic review	☐	☐	NA
Update	1b	If the protocol is for an update of a previous systematic review, identify as such	☐	☐	NA
**Registration**	2	If registered, provide the name of egistry (e.g., PROSPERO) and tration number in the Abstract	√☐	☐	CRD42022364964
**Authors**					
Contact	3a	Provide name, institutional ıtion, and e-mail address of all ›col authors; provide physical mailing ess of corresponding author	√☐	☐	1
Contributions	3b	Describe contributions of protocol ›rs and identify the guarantor of the w	√☐	☐	15
**Support**					

Section/topic	#	Checklist item	Information rted	Page ber(s)	
			Yes	No	
Sources	5a	Indicate sources of financial or other ort for the review	√☐	☐	15
Sponsor	5b	Provide name for the review funder or sponsor	☐	☐	NA
Role of sor/funder	5c	Describe roles of funder(s), sor(s), and/or institution(s), if any, in loping the protocol	☐	☐	NA
**ABSTRACT** Structured mary	6	Provide a structured summary: ground, objectives, methods, lts, conclusions and implications of findings	√☐	☐	2
**INTRODUCTION**					
**Rationale**	8	Describe the rationale for the review e context of what is already known	√☐	☐	4-5
**Objectives**	8	Provide an explicit statement of the tion(s) the review will address with ence to participants, interventions, parators, and outcomes (PICO)	√☐	☐	5
**METHODS**					
**Eligibility criteria**	9	Specify the study characteristics PICO, study design, setting, time e) and report characteristics (e.g., considered, language, publication s) to be used as criteria for eligibility he review	√☐	☐	5

Section/topic	#	Checklist item	Information rted	Page ber(s)	
			Yes	No	
**Information sources**	10	Describe all intended information :es (e.g., electronic databases, contact study authors, trial registers, or other literature sources) with planned dates verage	√☐	☐	3-4
**Search strategy**	11	Present draft of search strategy to ɪed for at least one electronic ɔase, including planned limits, such t could be repeated	√☐	☐	5
STUDY RECORDS					
Data management	12a	Describe the mechanism(s) that will ɪed to manage records and data ɪghout the review	√☐	☐	6
Selection process	12b	State the process that will be used electing studies (e.g., two ɔendent reviewers) through each e of the review (i.e., screening, ɪility, and inclusion in meta-analysis)	√☐	☐	6
Data collection ess	12c	Describe planned method of cting data from reports (e.g., piloting s, done independently, in duplicate), ɔrocesses for obtaining and confirming from investigators	√☐	☐	5-6
**Data items**	13	List and define all variables for ı data will be sought (e.g., PICO items, ng sources), any pre-planned data ɪptions and simplifications	√☐	☐	5

Section/topic	#	Checklist item	Information rted	Page ber(s)	
			Yes	No	
**Outcomes and itization**	14	List and define all outcomes for ı data will be sought, including itization of main and additional ɔmes, with rationale	√☐	☐	5
**Risk of bias in individual es**	15	Describe anticipated methods for ısing risk of bias of individual studies, ding whether this will be done at the ɔme or study level, or both; state how nformation will be used in data ıesis	√☐	☐	6
DATA					
**Synthesis**	16a	Describe criteria under which study will be quantitatively synthesized	√☐	☐	6
	16b	If data are appropriate for titative synthesis, describe planned nary measures, methods of handling and methods of combining data from es, including any planned exploration nsistency (e.g., I^2, Kendall's tau)	√☐	☐	NA
	16c	Describe any proposed additional ɪses (e.g., sensitivity or subgroup ɪses, meta-regression)	√☐	☐	NA
	16d	If quantitative synthesis is not ɔpriate, describe the type of summary ıed	√☐	☐	6

Section/topic	#	Checklist item	Information rted	Page ber(s)	
			Yes	No	
**Meta-bias(es)**	17	Specify any planned assessment of -bias(es) (e.g., publication bias across es, selective reporting within studies)	√☐	☐	NA
**Confidence in ılative evidence**	18	Describe how the strength of the of evidence will be assessed (e.g., ÞE)	√☐	☐	NA
**RESULTS**					
Study selection	19	Give numbers of studies screened, ;sed for eligibility, and included in the w, with reasons for exclusions at each ;, ideally with a flow diagram.	√☐	☐	6
Study characteristics	20	For each study, present ıcteristics for which data were cted (e.g., study size, PICOS, follow-eriod) and provide the citations.	√☐	☐	6
Risk of bias within es	21	Present data on risk of bias of each / and, if available, any outcome level ;sment (see item 12).	√☐	☐	6
Results of individual es	22	For all outcomes considered efits or harms), present, for each /: (a) simple summary data for each vention group (b) effect estimates and dence intervals, ideally with a forest	√☐	☐	Supplementary 4a-4b
Synthesis of results	23	Present results of each meta-ysis done, including confidence vals and measures of consistency.	√☐	☐	7-11, Table 1

Section/topic	#	Checklist item	Information rted	Page ber(s)	
			Yes	No	
Risk of bias across es	24	Present results of any assessment of ıf bias across studies (see Item 15).	√☐	☐	NA
Additional analysis	25	Give results of additional analyses, if (e.g., sensitivity or subgroup ses, meta-regression [see Item 16]).	☐	√☐	NA
**DISCUSSION**					
Summary of evidence	26	Summarize the main findings ding the strength of evidence for each outcome; consider their relevance to roups (e.g., healthcare providers, , and policy makers).	√☐	☐	13
Limitations	27	Discuss limitations at study and ome level (e.g., risk of bias), and at w-level (e.g., incomplete retrieval of ified research, reporting bias).	√☐	☐	14
Conclusions	28	Provide a general interpretation of esults in the context of other nce, and implications for future rch.	√☐	☐	14
**FUNDING**					
Funding	29	Describe sources of funding for the matic review and other support (e.g., ly of data); role of funders for the matic review.	√☐	☐	10

Supplementary Table 2: Full searching strategy by databases [date: 18-19/10/2022]; updated on 23/01/2023

Searching terms for concepts (a) conflict, b(health), and (c) Tigray

Conflict	Health	Tigray
assault*	health	Tigray
arrest	health impact*	Tigrai
detain	health outcome	Tigrayan
kidnap*	*care*	north* Ethiopia
Torture	health care	ethiopia
Attack	Healthcare	
shoot	medical care	
stab*	public health	
threat	health facility*	
violence	Facility	
abuse	health infrastructure	
seig	health post	
loot	health centre	
obstruct*	health center	
occupy	Hospital	
protect	medical clinic	
conflict	clinic, medicine	
terror*	Pharmacy	
bomb*	Ambulance	
airstrike	medical transport	
war*	Transport	
armed conflict	medical facility	
armed	access	
emergency	Block	
fragile state	patient*	
uprising	Ill	
perilous	sick	
	wounded	
	medicin*	

	medic*	
	medical personnel	
	health personnel	
	health staff	
	health worker	
	health professional	
	physician	
	doctor*	
	surgeon	
	nurse*	
	gp	

Medline/ Ovid MEDLINE(R) searching strategy and results [Date of Search run 18/10/2022]

#	Query	Results
1	conflict.mp.	80,323
2	Violence/ or assault.mp. or Crime Victims/	53,630
3	violence.mp. or Gender-Based Violence/ or Violence/	77,567
4	Terrorism/ or Bombs/ or bombing.mp.	6,682
5	war.mp. or Armed Conflicts/	54,161
6	seige.mp.	18
7	Health/eh, st, sn, td [Ethnology, Standards, Statistics & Numerical Data, Trends]	317
8	health facility.mp. or Health Facilities/	44,368
9	transport.mp. or Transport Vesicles/	670,861
10	health care.mp. or "Delivery of Health Care"/	902,602
11	health care.mp.	902,602
12	patient.mp. or Patients/	3,149,476
13	Refugees/ or humanitarian.mp. or Warfare/ or Disasters/	60,086
14	Ethiopia/ or Tigray.mp.	18,038
15	Ethiopia/ or tigrai.mp.	17,846

16	North Ethiopia.mp.	134
17	Tigrayan.mp.	7
18	14 or 15 or 16 or 17	18,072
19	arrest.mp.	170,737
20	"Emigrants and Immigrants"/ or detain.mp.	14,947
21	kidnap*.mp.	407
22	torture.mp. or Torture/	3,073
23	attack.mp.	106,990
24	shoot.mp.	27,460
25	Wounds, Stab/ or Wounds, Gunshot/ or stab*.mp.	1,424,196
26	Humans/ or threat.mp.	20,845,270
27	abuse.mp.	188,495
28	Humans/ or loot*.mp.	20,805,859
29	Humans/ or obstruct*.mp.	20,870,513
30	Humans/ or occup*.mp.	20,923,775
31	Humans/ or protect.mp.	20,889,449
32	bomb*.mp.	38,702
33	airstrike.mp.	5
34	armed.mp. or Humans/	20,810,920
35	emergency.mp. or Emergencies/	394,674
36	Warfare/ or fragile state.mp. or Health Services Accessibility/	106,981
37	uprising.mp.	401
38	Humans/ or perilous.mp.	20,806,015
39	1 or 2 or 3 or 4 or 5 or 6 or 19 or 20 or 21 or 22 or 23 or 24 or 25 or 26 or 27 or 28 or 29 or 30 or 31 or 32 or 33 or 34 or 35 or 36 or 37 or 38	22,142,143
40	Public Health/ or health impact*.mp.	109,133
41	Humans/ or health outcome.mp.	20,806,479
42	healthcare.mp.	337,668
43	Humans/ or medical care.mp.	20,816,362
44	Health Facilities/ or health facilit*.mp. or Health Facility Environment/	56,626

45	Health Services Accessibility/ or facility.mp.	198,261
46	health infrastructure.mp.	1,622
47	Community Health Workers/ or health post.mp.	6,850
48	Primary Health Care/ or health center.mp.	101,245
49	Primary Health Care/ or health centre.mp.	94,445
50	hospital.mp. or Hospitals/	1,482,306
51	Primary Health Care/ or medical clinic.mp.	90,905
52	clinic.mp. or Humans/	20,857,629
53	medicine.mp. or Medicine/	970,029
54	Pharmacy/ or pharmacy.mp.	72,521
55	ambulance.mp. or Ambulances/	14,446
56	Humans/ or "Transportation of Patients"/ or medical transport.mp. or "Wounds and Injuries"/ or Emergency Medical Services/	20,818,531
57	medical facility.mp.	1,374
58	access.mp.	405,517
59	block*.mp.	921,075
60	ill.mp.	153,751
61	sick.mp.	50,262
62	Military Medicine/ or "Wounds and Injuries"/ or wounded.mp.	116,214
63	Emergency Medical Technicians/ or Military Personnel/ or Military Medicine/ or "Wounds and Injuries"/ or medic*.mp. or Emergency Medical Services/	3,297,893
64	"Delivery of Health Care"/ or medical personnel.mp. or Health Personnel/	171,914
65	health personnel.mp. or Health Personnel/	201,705
66	health staff.mp. or Health Personnel/	62,175
67	Community Health Workers/ or health worker.mp. or Health Personnel/	68,579
68	physician.mp. or Physicians/	348,622
69	doctor*.mp. or Physicians/	227,852
70	surgeon.mp. or Surgeons/	114,151
71	nurse.mp. or Nurses/	223,085
72	General Practice/ or General Practitioners/ or GP.mp.	65,529

73	40 or 41 or 42 or 43 or 44 or 45 or 46 or 47 or 48 or 49 or 50 or 51 or 52 or 53 or 54 or 55 or 56 or 57 or 58 or 59 or 60 or 61 or 62 or 63 or 64 or 65 or 66 or 67 or 68 or 69 or 70 or 71 or 72	22,658,579
74	18 and 39 and 73	15,578
75	limit 74 to (english language and humans and yr="2020 -Current")	4,783

Pub med searching strategy and results [Date of Search run 18/10/2022]

#	Query	Results
1	((((((((((((((((((((((((((("conflict"[All Fields])) OR ("arrest"[All Fields])) OR ("detain"[All Fields])) OR ("kidnap"[All Fields])) OR ("torture"[All Fields])) OR ("attack"[All Fields])) OR (shoot)) OR ("stab"[All Fields])) OR ("threat"[All Fields])) OR ("violence"[All Fields])) OR ("abuse"[All Fields]))) OR ("siege"[All Fields])) OR ("loot"[All Fields])) OR ("obstruct"[All Fields])) OR ("occupy"[All Fields])) OR ("protect"[All Fields]))) OR ("terror and disaster"[All Fields])) OR ("bombing"[All Fields])) OR ("airstrike"[All Fields])) OR ("war"[All Fields])) OR ("armed conflict"[All Fields])) OR ("armed"[All Fields])) OR ("emergency"[All Fields])) OR ("fragile"[All Fields])) OR ("uprising"[All Fields]))) OR ("perilous"[All Fields])	2,707,609
2	((((((((((((((((((((((((((((((((((("health"[All Fields])) OR ("health impact"[All Fields])) OR ("health outcome"[All Fields])) OR ("health care"[All Fields])) OR ("healthcare"[All Fields])) OR ("medical care"[All Fields])) OR ("public health"[All Fields])) OR ("health facility"[All Fields])) OR ("facility"[All Fields])) OR ("health infrastructure"[All Fields])) OR ("health post"[All Fields])) OR ("health center"[All Fields])) OR ("health centre"[All Fields])) OR ("hospital"[All Fields])) OR ("medical clinic"[All Fields])) OR ("pharmacy"[All Fields])) OR ("ambulance"[All Fields])) OR ("medical transport"[All Fields])) OR ("transport"[All Fields])) OR ("medical facility"[All Fields])) OR ("access"[All Fields])) OR ("block"[All Fields])) OR ("patient"[All Fields])) OR ("ill"[All Fields])) OR ("sick"[All Fields])) OR ("wounded"[All Fields])) OR ("medicine"[All Fields])) OR ("medical personnel"[All Fields])) OR ("health personnel"[All Fields])) OR ("health staff"[All Fields])) OR ("health worker"[All Fields])) OR ("health professional"[All Fields])) OR ("physician"[All Fields])) OR ("doctor"[All Fields])) OR ("surgeon"[All Fields])) OR ("nurse"[All Fields])) OR ("gp"[All Fields])	15,864,159
3	(((((("tigray"[All Fields])) OR ("tigrai"[All Fields])) OR ("tigrayan"[All Fields])) OR ("north ethiopia"[All Fields])) OR ("northern ethiopia"[All Fields])) OR ("ethiopia"[All Fields])	33,970
4	1 AND 2 AND 3; limited with year (2020), species (human), language (English)	1,432

Scopus searching strategy and results [Date of Search run 18/10/2022]

#	Query	Results
1	TITLE (*conflict* OR *arrest* OR *detain* OR *kidnap** OR *torture* OR *attack* OR *shoot* OR *stab** OR *threat* OR *violence* OR *abuse* OR *sieg** OR *loot* OR *obstruct** OR *occupy* OR *protect* OR *terror** OR *bomb** OR *airstrike* OR *war** OR *"armed conflict"* OR *armed* OR *emergency* OR *"fragile state"* OR *uprising* OR *perilous*)	1,881,704
2	TITLE (health OR "health impact*" OR "health outcome" OR "health care" OR healthcare OR "public health" OR "health facility" OR facility OR "health infrastructure" OR "health post" OR "health centre" OR "health center" OR hospital OR "medical clinic" OR pharmacy OR ambulance OR "medical transport" OR transport OR "medical facility" OR access OR block OR patient OR ill OR sick OR wounded OR "medical personnel" OR " health personnel" OR "health staff" OR "health worker" OR "health professional" OR physician OR doctor OR surgeon OR nurse OR gp)	4,984,260
3	TITLE (*tigray* OR *tigrai* OR *tigrayan* OR *"north*ethiopia"* OR *ethiopia*)	32,211
4	1 AND 2 AND 3; limited to year (2020-2022) and language (English)	119

CINAHL Searching strategy and results [Date of Search run 19/10/2022]

No	Search Strategy	Results
S1	TI conflict OR TI (assault or violence or aggression) OR TI (arrests or incarceration or conviction) OR TI detain OR TI kidnap OR TI torture OR TI attack OR TI shoot OR TI stabbing OR TI (threat or risk or danger) OR TI abuse OR TI siege	352,463
S2	TI loot OR TI obstruction OR TI occupy OR TI protect OR TI terrorist attacks OR TI terrorism OR TI terrorist OR TI bombing OR TI bomb OR TI airstrikes OR TI war OR TI war crimes	28,880
S3	TI armed conflict OR TI (armed forces or military) OR TI armed OR TI (armed conflict or war) OR TI armed robbery OR TI (emergency department or emergency room or accident and emergency or accident & emergency or a&e or a & e) OR TI fragile states OR TI uprising	55,798
S4	S1 OR S2 OR S3	424,664
S5	TI health OR TI (healthcare or health care or hospital or health services or health facilities) OR TI health promotion OR TI healthcare workers OR TI healthcare professionals OR TI (health impacts or health effects or health risks or health implications) OR TI (health outcomes or health consequences or health impacts or health effects) OR TI medical care OR TI medical devices OR TI (public health or community health or population health) OR TI facility services OR TI health infrastructure	620,302
S6	TI (health center or health centre) OR TI medical clinic OR TI (pharmacy or pharmacies or pharmacist or pharmacists) OR TI (ambulance or ambulance service or paramedic or emergency medical service or ems or pre-hospital or prehospital) OR TI medical transport OR TI (medical facility or hospital or clinic) OR TI (access to care or access to healthcare or access to services) OR TI block OR TI patient OR TI (illness or disease) OR TI sick OR TI wounded	1,202,660
S7	TI (medical personnel or health personell) OR TI health staff OR TI health staffing shortage OR TI (physicians or doctors or clinicians or healthcare professionals) OR TI (doctors or physicians or healthcare professionals) OR TI (surgeons or physicians or doctors) OR TI (nurse practitioner or advanced practice nurse or apn or np)	112,053
S8	S5 OR S6 OR S7	1,711,912
S9	TI tigray OR TI tigray war OR TI tigray crisis OR TI tigray conflict OR TI north ethiopia OR TI tigrai OR TX ethiopia	9,654
S10	S4 AND S8 AND S9	185
S11	S4 AND S8 AND S9; limited to year 2020-2022	81

Web of Science searching strategy and results [Date of Search run 19/10/2022]

No	Search Strategy	Results
1	conflict Or assault* Or arrest Or detain Or kidnap* Or torture Or attack Or shoot Or stab* Or threat Or violence Or abuse Or *sieg* Or loot Or obstruct* Or occupy Or terror* Or bomb* Or airstrike Or war* Or "armed conflict" Or armed Or emergency Or "fragile state" Or uprising Or perilous	7,850,110
2	health Or "health impact*" Or "health outcome" Or *care* Or "health care" Or healthcare Or "medical care" Or "public health" Or "health effects" Or "health consequence" Or "health service" Or "health promotion" Or "health facility" Or "health infrastructure" Or "health post" Or "health centre" Or "health center" Or hospital Or "medical clinic" Or pharmacy Or ambulance Or "medical transport" Or "medical facility" Or access Or block Or sick Or ill Or medic* Or "medical personnel" Or "health personnel" Or "health staff" Or "health worker" Or "health professional" Or physician Or doctor* Or surgeon Or nurse*Or Gp	9,512,603
3	tigray Or tigrai Or tigrayan Or "north* ethiopia" Or ethiopia	44,316
4	1 And 2 And 3; limiting by year (2020 onwards) and language (English)	1,622

Supplementary Table 3a. JBI quality appraisal outputs for commentary studies and letters (n=27)

Covidence #	Source	Field of expertise	Population of interest	position and logic	Reference/s	Incongruence	Total score (out of 6)	Overall appraisal
6311	Yes	Yes	Yes	Yes	Yes	Yes	6	Include
6310	Yes	Yes	Yes	Yes	Yes	Yes	6	Include
6309	Yes	Yes	Yes	Yes	Yes	Yes	6	Include
6306	Yes	Yes	Yes	Yes	Yes	Yes	6	Include
6301	Yes	Yes	Yes	Yes	Yes	Yes	6	Include
6300	Yes	Yes	Yes	Yes	No	Yes	6	Include
6299	Yes	Yes	Yes	Yes	Yes	Yes	6	Include
5836	Yes	Yes	Yes	Yes	Yes	Yes	6	Include
5835	Yes	Yes	Yes	Yes	Yes	Yes	6	Include
5790	Yes	Yes	Yes	Yes	Yes	Yes	6	Include
5358	Yes	Yes	Yes	Yes	Yes	Yes	6	Include
4567	Yes	Yes	Yes	Yes	Yes	Yes	6	Include
4305	Yes	Yes	Yes	Yes	Yes	Yes	6	Include

3825	Yes	No	Yes	No	Yes	Yes	4	Include
1981	Yes	No	Yes	No	Yes	Yes	4	Include
1980	Yes	No	Yes	No	Yes	Yes	4	Include
1979	Yes	No	Yes	No	Yes	Yes	4	Include
1978	Yes	No	Yes	No	Yes	Yes	4	Include
1977	Yes	No	Yes	No	Yes	Yes	4	Include
1635	Yes	No	Yes	No	Yes	Yes	4	Include
1622	Yes	Yes	Yes	Yes	Yes	Yes	6	Include
1507	Yes	No	Yes	Yes	Yes	Yes	4	Include
6299	Yes	No	Yes	Yes	Yes	Yes	4	Include
6318	Yes	Yes	Yes	Yes	Yes	Yes	6	Include
6315	Yes	Yes	Yes	Yes	Yes	Yes	6	Include
6317	Yes	Yes	Yes	Yes	Yes	Yes	6	Include

Supplementary Table 3b. JBI quality appraisal outputs for original article studies and reports (n=12)

Covidence #	Sample criteria	Study subject & setting	Valid & reliable exposure measurement	Objective outcome condition	Confounding identified	dealing confounding	Valid & reliable outcome measurement	Appropriate analysis	Total score (out of 8)	Overall appraisal
6307	Yes	Yes	Yes	Yes	Yes	Yes	Yes	Yes	8	Include
6305	No	Yes	Yes	No	No	No	No	Unclear	2	Include
6304	Yes	Yes	Yes	Yes	No	NA	Yes	Yes	6	Include
6303	NA	Yes	Yes	Yes	NA	NA	Yes	Yes	5	Include
6302	No	Yes	Yes	Yes	No	No	Yes	Yes	5	Include
4591	NA	Yes	Yes	Yes	NA	NA	Yes	Unclear	4	Include
2671	NA	Yes	Yes	Yes	NA	NA	Yes	Yes	5	Include
2486	Yes	Yes	Yes	Yes	Unclear	Unclear	Yes	Yes	6	Include
1293	Yes	Yes	Unclear	Yes	Unclear	Unclear	Unclear	Yes	4	Include
6297	Yes	Yes	Yes	Yes	Unclear	Unclear	Yes	Yes	6	Include
6316	Yes	Yes	Yes	Yes	Unclear	Unclear	Yes	Yes	6	Include
6314	Yes	Yes	Yes	Yes	Yes	Yes	Yes	Yes	8	Include

NA= Not applicable

Supplementary Table 3c. JBI quality appraisal outputs for case studies (n=2)

Covidence #	Patient demography	Patient history	Current clinical condition	diagnostic test	treatment procedure	Post-clinical activity	Adverse event	case report	Total score (out of 8)	Overall appraisal
1292	Yes	Yes	Yes	Yes	No	Yes	Yes	Yes	7	Include
6308	Yes	Yes	Yes	Yes	No	Yes	Yes	Yes	7	Include

NA= Not applicable

Supplementary Table 4a. Summary of key issues addressed in documents included in the review on conflict and health in Tigray, 2020-2022

1^0 Author et al, Year	Facilities and personnel	Health/social care services	Outcomes or impacts	Responses, rebuilding, and recommendations
Commentaries[1-24]				
Clarfield et al, 2022[16]	• Absence of salary/access to savings of HWs	• Reduction of essential medication availability<br>• Blockade of essential medications		• Call to UN, AU, WHO, Nephrology organizations to stand by patients & health workers
Burki, 2022[3]	• Absence of salary/access to savings of HWs & aid workers<br>• Attack on ambulances	• Collapse of Covid-19 Services<br>• Lack of basic medications for diabetes, HIV, TB, malaria, scabies patients<br>• Lack of malnutrition & reproductive health kits<br>• Lack of food for admitted HIV/other patients<br>• Cut telephone lines<br>• Siege on Tigray by Federal government<br>• Blocking aid workers and organizations	• Reduced childhood vaccination rates<br>• Notifications of outbreaks<br>• Lack of access to water<br>• TB/HIV lost to follow up<br>• Increased malnutrition<br>• Food insecurity<br>• Mass killings<br>• Gang-rape<br>• violations of international humanitarian	• Scabies is being treated with expired permethrin
Berhe et al, 2022[1]	• Health infrastructure damage	• Dialysis service stoppage<br>• Reduced dialysis frequency to patients	• Mortality of patients received dialysis<br>• Vicarious trauma to health workers	• Using expired central venous catheters or exchanging dialyzers<br>• Use of locally prepared fluids to treat Peritoneal dialysis<br>• Call to lift blockade and resume services
Berhe et al, 2022[2]	• Health infrastructure damage/looting<br>• Absence of salary/access to savings of HWs	• Interruption of haemodialysis services<br>• Stoppage of health extension program<br>• Lack of transportation and communication blackouts<br>• Shortage of dialysis supplies and lack of available basic medications	• Loss to follow up of patients<br>• Death of patients<br>• Vicarious trauma to health workers (HWs)	• Call to full resumption of access to health to the besieged Tigray<br>• Call to mental health service provision to HWs<br>• Call to stop conflict

1[0] Author et al, Year	Facilities and personnel	Health/social care services	Outcomes or impacts	Responses, rebuilding, and recommendations
Hiluf et al, 2022[4]	• Destruction of health facilities<br>• Blockade of cash/money, transportation, and communication	Medieval type of siege	• vicarious trauma of health workers	• Use of expired drugs to treat patients<br><br>Call to international community to fairly treat/advocate Tigray-Ukraine war.
Cousins S, 2022[5]	• Health infrastructure destruction		• Increase fistula cases<br>• Death and displacement of civilians<br>• Gang rape	
Gebregziabher et al, 2022[6]	• Health care system destroyed<br>• Ambulance attacked<br>• Internal displacement/not reporting of health workers	• extreme lack of food<br><br>deliberately blocked humanitarian by federal government	• Rape of women and girls<br>• extreme lack of food	
Kumar et al, 2022[7]	• Destruction of health facilities<br>• Looting of ambulances<br>• Killing & displacement of health workers	• Non-functionality of health facilities<br>• Non-functionality of the only oxygen-producing company in Tigray<br>• lack of medication and other essential medical supplies for NCDs and other patients	• Malnutrition of children and breast-fed/pregnant mothers<br>• Weaponization of rape<br>• Death and hunger<br>•	• Comprehensive physical and mental health care for patients and health workers<br>• Provision of policy expertise and funding to rebuild the healthcare infrastructure in Tigray.<br>• businesses and community members have donated soap, linen, and bedsheets to sustain hospital operations<br>• Call for Ethiopian government to allow unfettered humanitarian aid and in priority<br><br>Call for lobal health academics and international aid organizations to advocate
Wall L, 2022[8]	• Deliberate destruction of health facilities<br>• Harassment of aid/health workers	• Non-functionality of health facilities<br>• Lack of basic medical supplies<br>•	• Weaponization of rape<br>• extrajudicial executions<br>• displacement<br>• Starvation of doctors	• Call for international community to condemn medical siege in Tigray

1[0] Author et al, Year	Facilities and personnel	Health/social care services	Outcomes or impacts	Responses, rebuilding, and recommendations
Makoni M, 2022[9]	• Destruction of health facilities	• unavailability of power, water, and essential devices<br>• Non-functionality of health facilities<br>• Closure of cancer clinic<br>• delaying permission by Ethiopian government for aid agencies to deliver service in Tigray		• Patients with terminal disease were being treated with expired medication and paracetamol
Paltiel O et al, 2022[10]	• Doctors in Tigray are unpaid and malnourished		• Malnutrition of children increased<br>• Mortality of caner and dialysis patients<br>• Food insecurity increase	• show solidarity to health workers
Tesema et al, 2021[11]	• Destruction of health facilities<br>• Killing of health workers (MSF)	• Non-existent of health care structure	• women and girls were subjected to sexual abuse<br>• Displacement & starvation in millions	• Focus area of rebuilding and reorienting collapsed healthcare system
Teka et al, 2022[12]	• Destruction of health facilities	• Collapsed health care system<br>• Total blockade of humanitarian aid, communication, power, and banking services<br>• Running out of chemotherapy drugs, screening and diagnostic supplies, stoppage of surgery<br>• lack supplemental oxygen, anesthetic medications, antibiotics, operation theater materials, and fuel to run generators	• Siege on the entire population<br>• Normalization of ddisplacement, death, and destruction	• Use of expired chemotherapy as a remedy<br>• Calling the international community, cancer societies, humanitarian organizations, and the UN agencies to advocate the manmade catastrophe in Tigray and enforcing to end the conflict
Gesesew et al, 2022[13]	• Health care system destroyed<br>• Ambulance attacked	• Non-functionality of health facilities<br>• extreme lack of food<br>• deliberately blocked humanitarian by federal government<br>• lack of basic medications	• Women and girls are suffering from Fistula<br>• No treatment to get treatment due to cost of transport and lack of access to healthcare<br>• Risk factors to fistula among women and girls in Tigray included obstructed labor, malnutrition, gang-rape, & sexually transmitted infections.	

1[0] Author et al, Year	Facilities and personnel	Health/social care services	Outcomes or impacts	Responses, rebuilding, and recommendations
			• Reduction of antenatal care and skilled delivery<br>• Increased acute malnutrition among pregnant and lactating mothers.<br>• Deliberate transmission of STIs	
Hadera et al, 2022[14]	• Hospital attacks by drones or jets	• Vicarious trauma of health workers<br>• No salary to health workers<br>• Lack of enough medications, supplies and emergency services to patients including oxygen and basic test kits.<br>• indiscriminate airstrikes<br>• Blockade of NGOs who provide health services<br>• Cancelation of 3700 and above surgeries due to a lack of oxygen in 12 months.	• spike in death rates<br>• drop in the quality of healthcare services at hospitals<br>• deaths of more than 183 neonates in the past six months and 31% of patients admitted to the ICU.<br>• Mortality rate at the emergency department increased from the 0.3% pre-war level to 2.5% at a regional level.<br>• unsafe blood transfusion as blood banks have run out of testing kits to check for HIV, hepatitis, syphilis	
Yemane et al, 2022[15]	• Deliberate destruction of health facilities	• Blockade of food and medical supplies<br>• No basic health service provision	• Massacred, displacement<br>• Gang rape<br>• Malnutrition, food insecurity	• Re-washing gloves as a intervention<br>• Call to lift siege
Devi S, 2022[17]	• Health infrastructure destruction	• Lack of essential medical materials e.g. glove<br>• Systematic blockade of food, medication<br>• Systematic blockade of tele, fuel, cash	• Death and starvation of civilians<br>• Increased malnutrition of children and pregnant and breastfeeding women	• ICRC resumed cargo flights on Jan 26, 2022, delivering supplies, including insulin, oxytocin, tetanus toxoid, gloves, and surgical materials on 11 Feb 2022
Devi S, 2021[18]	• Road blocks and military restrictions	• Deliberate blockade of food and supplies by Eritrean military	• Imminent risk of famine<br>• Displacement<br>• Expected raise of malaria<br>• reduction in child vaccination	

1[0] Author et al, Year	Facilities and personnel	Health/social care services	Outcomes or impacts	Responses, rebuilding, and recommendations
Devi S, 2021[19]	• Health infrastructure damage/looting<br>• Threatening of UN aid workers staff by airstrike	• Suspension of MSF & NRC (global NGO health service providers)	• Killing of civilians<br>• Increased malnutrition of children and pregnant and breastfeeding women<br>• No vaccination<br>• Increased malaria, dysentery, & rabies cases	
Devi S, 2021[20]	• Health infrastructure damage<br>• MSF marked convoy blocked by Ethiopian military	• Systematic blockade of food<br>• Health facilities non-functionality	• Mass killing/ extrajudicial killings<br>• Gang-rape<br>• Ethnic cleansing<br>• NCD patients without pills	
Devi S, 2020[21]		• lack of sutures, antibiotics, painkillers, gloves<br>• reduction of consultations<br>• suspension of services to treat injuries<br>• blockade of aid trucks by Ethiopian officials	• trauma increased	
Mulugeta et al, 2022[22]	Destruction of health facilities	total blockade of humanitarian aid,	• Malnutrition of children increased<br>• Household food security increased<br>• Displacement of people in millions	
Teka et al, 2022[23]	• Targeted destruction of health facilities<br>• looting of ambulances<br>• blocking civilian patients at checkpoints not get access services	• no chemotherapy drugs allowed in to Tigray<br>• diagnostic procedures and tests are no longer	• Death<br>• Rape weaponization<br>• Displacement & starvation in millions	Call for AU, UN, and others to press for unfettered humanitarian access
Weldegerima et al, 2022[24]	• Deliberate destruction of health facilities<br>• Harassment of aid/health workers	• Collapsed health care system<br>• Lack of basic medical supplies	• Weaponization of rape<br>• extrajudicial executions<br>• displacement<br>• vicarious trauma of practitioners	Call to cardiology communities to allow medical supplies to cardiac and other patients by the Ethiopian government

1[0] Author et al, Year	Facilities and personnel	Health/social care services	Outcomes or impacts	Responses, rebuilding, and recommendations
Original articles[25-33]				
Gesesew et al, 2021[33]	• deliberate attack on (weaponization of) health infrastructure<br>• Attack on ambulance<br>• Unknown status of HEWs<br>• No reporting back of health workers to workplace<br>•	• Collapse in MCH health service<br>• HEP non-functionality<br>• Woreda health office non-functionality	• Weaponization of health care<br>• Increased malnutrition<br>• Fear of cholera and vaccine preventable disease outbreaks<br>• Gang rape<br>• Food insecurity<br>• Displacement	• Co-operative international push to stop the collective punishment of Tigray should be of the highest priority in order to open up basic public services including healthcare<br>• Health workers around the glob should be voice for the health workers in the war-torn Tigray
Berhe et al, 2022[25]		• interrupted haemodialysis sessions	• Mortality of dialysis patients	• Call to Ethiopian government to allow basic amenities<br>• Call to UN, AU, WHO, Nephrology organizations to stand by patients & health workers
Favara et al, 2022[26]			• Allegation of war crimes<br>• Increased depression, anxiety (by gender)	
Gebregziabher et al, 2022[27]	• Attack on health infrastructure by zones	• Health facilities non-functionality		• War as a public health priority
Hailu et al, 2022[28]		• NCD follow up interruption<br>• West Tigray, private health institutions and other referral institutions not included in study setting (data set unavailable)		
Weledegebriel et al, 2022[29]		• Declining HIV services, clinical follow-up, laboratory service, and ART	• Reduction of HIV patients follow up	
Abay et al, 2022[30]		• sharply increment of households	• inability to buy enough food, teff, wheat, maize, oil	

1⁰ Author et al, Year	Facilities and personnel	Health/social care services	Outcomes or impacts	Responses, rebuilding, and recommendations
		who were unable to access health services or buy medicines<br>• reduction of households' ability to buy medicine<br>• reduction of households' ability to access enough soap		
Phillips et al, 2022[31]	• Destruction of health facilities			• Use Health extension program (HEP) approach to address the collapsed healthcare system
Abebe et al, 2022[32]	• Health infrastructure damage<br>• Attrition & displacement of health workers<br>• Blockade of volunteer diaspora health workers	• Blockade of fuel<br>• reduction of households' ability to buy medicine<br>• reduction of households' ability to access enough soap	• Increased SGBV<br>• Weaponization of rape<br>• inability to buy enough food	• Training to local leaders, influencers and institutions to support rape survivors
Reports[34-36]				
HRW, 2022[34]	• Destruction of health facilities<br>• armed groups threatened, harassed, or detained Tigrayan aid worker<br>• threats and intimidation of health workers and humanitarian aid providers in Tigray<br>• blocking rape survivors at checkpoints not to get access services	• Non-functionality of health facilities<br>• denying desperately needed food, medical supplies and fuel; and banking, electricity, and telecommunications to the population of Tigray	• Weaponization of rape (of 6 to 80 years old)<br>• numerous grave abuses, including massacres	• Mental health care and psychosocial support services for patients and health workers<br>• Call for safe, and unfettered access to humanitarian aid
TRHB, 2022[35]	• Deliberate destruction of health facilities<br>• No salary/access to bank for health workers for almost two years<br>• Killing and displacement of health workers<br>• Attack on ambulances	• to lack of basic drug supplies, medical equipment, and operational problems<br>• Total blockade of entire Tigray population	• Famine<br>• Violations of Geneva convention<br>• Missing health facilities visits and vaccination<br>• Acute malnutrition among Pregnant and Lactating Women<br>• Displacement and mortality of civilians, including mass killings	• Some vaccination and mobile health clinic programs started with the help of NGOs

1⁰ Author et al, Year	Facilities and personnel	Health/social care services	Outcomes or impacts	Responses, rebuilding, and recommendations
			• Mortality/lost to follow up of chronic case patients	
WFP, 2022[36]			• Malnutrition of children, pregnant and breast-feeding mothers<br>• Food insecurity	• Field work challenges to conduct face-to-face survey due to security reasons.
Letters[37-39]				
Hiluf et al, 2022[37]	• Destruction of health facilities<br>• Blockade of cash/money, transportation, and communication	• Near-total collapse of health system in the region<br>• lack of reagents, not functioning of CT Scan, MRI and mammography	• Displacement, and death as normal elements of war<br>• loss of therapeutic and survival benefits of cancer patients<br>• vicarious trauma of health workers	• Use of expired chemotherapy as an option<br>• Call to oncology association to raise their voices re the collapsed cancer care in Ayder, Federal MoH to support essential medical supplies for cancer patients, Global community to press to lift siege.
TRHB, 2022[38]		• Collapsed health care system<br>• No vaccination services<br>• Imposed siege/ defacto blockade	• Report of vaccine preventable illnesses outbreaks<br>• Reduction of vaccination coverage	• Calling GAVI to provide vaccine services by pressuring Federal government (to open corridor)
Hiluf et al, 2022[39]		• lack of insulin and other supplies	• developing complications such as DKA	• Calling to Ethiopian Diabetes Association to diabetic health care supplies and IV fluids.
Case studies[40 41]				
Berhe et al, 2022[40]		• interrupted kidney services		• Call to international nephrology societies and kidney transplant associations to advocate collapsed hemodialysis service
Gebrearegay et al, 2022[41]	• Killing of humanitarian aid<br>• Internal displacement/not reporting of health workers		• improvised equipment for peritoneal dialysis	• improvised equipment for peritoneal dialysis

Supplementary Table 4b: Main findings of each included document about conflict and health in Tigray, North Ethiopia, 2020-2022

1⁰ author et al, year	Document type	Population	Main findings
Abay et al, 2022	Original article		• share of households reporting inability to buy enough food increased from 5% in 2020 (just prior to the start of the war) to 29% in 2021.<br>• households who were unable to access health services or buy medicines had sharply increased<br>• the war reduced access by 35% points, while households' ability to buy medicine dropped by 8% points<br>• households' ability to access enough soap decreased by 16.6% points, however<br>• the patterns of demand for health services in Tigray did not change appreciably after the start of the war in November 2020<br>• the war caused no significant change in access to drinking or washing water
Abebe et al, 2022	Original article	General	• Health infrastructure has been severely damaged by the conflict through deliberate destruction, looting and even militant occupation of health facilities<br>• Only 27.5% of hospitals, 17.5% of health centres and none of 712 health posts were fully functional six months into the war.<br>• A lack of fuel in affected areas has also impeded the operation of Mobile Health Units.<br>• There were significant attrition and displacement of health workers<br>• Health workers in diasporic networks - such as a large roster maintained by the organisation HPN4Tigray - are eager to provide services directly. But they have been prevented from entering the country for fear of being arrested by the government as activists or terrorists.<br>• SGBV is widespread and used as a weapon of war. Training and support for local leaders, influencers and institutions to support survivors of widespread sexual and gender-based violence (SGBV) is critical.
Berhe et al, 2022	Letter to the Editor	People with chronic health condition (kidney)	• The war causes 70%-80% of health care facilities in the Tigray region dysfunctional<br>• War and blockade have caused reduced access, funding and severe supply shortages, including drastically curtailing of dialysis utilization<br>• Witnessing the painful deaths of veteran and new patients has imposed huge psychological burdens on the staff<br>• Overall, mortality in patients receiving hemodialysis in Ayder hospital increased from 25.5% (28 of 110 patients) in 2020 to 53.1% (43 of 81 patients) in 2021.<br>• Between July 1, 2021, and January 15, 2022, 61 new patients requiring emergency dialysis succumbed (including 36 with acute kidney injury) in Ayder hospital.<br>• 300 dialysis sessions (12.5%) entailed using single-use dialyzers for 6 to 8 times. Dialysis frequency was reduced from twice to once weekly or even fortnightly, impairing both quality of treatment and life.<br>• Using expired central venous catheters or exchanging dialyzers after patient deaths was among the desperate measures adopted<br>• Peritoneal dialysis was improvised using locally prepared fluids, saving a few lives. Currently, even intravenous fluid supplies have run out. Advising patients to stop dialysis and live out their remaining days untreated in hospital or at home is the tragic daily predicament of the trained dialysis team.<br>• The authors call upon nephrologists worldwide and relevant international bodies, including the ISN, to appeal to the Ethiopian Government to lift the blockade, re-establish supply chains, and restore health care, including the right to dialysis for the people of Tigray.

Berhe et al, 2022	Retrospective cohort study	patients on haemodialysis	• From the data available between 2013 and 2021, 550 patients received haemodialysis, of whom 181(32.7%) sufferedfromAKIand319(58%) suffered from chronic kidney disease (CKD). More than half of the patients have progressively succumbed to death following interrupted haemodialysis sessions leading to suboptimal haemodialysis.<br>• In 2021, during the war period, there were a total of 81 patients who underwent dialysis services; 69 (82.5%) of whom were newly enrolled patients in the same year. All of these patients have started dialysis on an emergency basis. Nearly half of the patients who are newly enrolled to the haemodialysis centre had only one haemodialysis session per week.<br>• The enrollment of patients in the haemodialysis service has decreased by 37.3% since the war broke out, in contrast to an annual increase by more than 30%.<br>• Mortality of patients under haemodialysis increased from 25.5% (28 deaths from 110 patients) in 2020 to 53% (43 deaths out of 81 patients) in 2021 ($p<0.05$). Similarly, patientâ€™s lost-to-follow-up increased from 9 to 14% (11/81 vs. 10/110) but was not statistically significant ($p=0.49$).<br>• Between July 1, 2021, and January 15, 2022, 61 new patients (36 AKI, including 6 patients with pregnancy related complications and 25 patients with CKD who needed emergency dialysis, succumbed owing to lack of haemodialysis supplies).<br>• The authors plead Ethiopian government to must allow the passage of life-saving medicines, essential medical equipment, and consumables for haemodialysis into Tigray.<br>• The authors demand that the relevant bodies including the United Nations, African Union, World Health Organization, Nephrology organizations, and, above all, the Ethiopian federal government (which has the primary responsibility) stand by the health care workers and the patients of Tigray.<br>• As nothing remains at the disposal of the staff caring for patients such as haemodialysis recipients, the international community and organizations with the capacity to help must organize funds to support Ayder Hospital

Berhe et al, 2022	Opinion	Health workers	• The hemodialysis center in Ayder hospital is currently unable to give appropriate care to kidney failure patients for a lack of access to dialysis supplies and consumables due to the ongoing war and siege.<br>• health facilities can no longer function, and 13% are partly damaged.<br>• Collapsed health care system and Stoppage of health extension program<br>• Hospitals across Tigray have been vandalised and looted, where 77% of the state<br>• Absence of salary and unable to access hard earned savings due to bank closure<br>• The fact Health workers unable to provide optimal haemolysis care has resulted in vicarious trauma of the health workers treating dialysis patients in the hospital and is manifested as follows: compassion fatigue, irritability, a feeling of bystander guilt; sadness about the patient's victimization, and hopelessness,<br>• financial hardship of patients, Lack of transportation and communication blackouts<br>• loss to follow up of patients<br>• Shortage of dialysis supplies and lack of available basic medications and interruption of haemodialysis service<br>• Deterioration of patient conditions, & Untimely death of patients<br>• Advocacy for a full resumption of access to health to the besieged Tigray,<br>• the provision of mental health service to health workers and special attention to health healthcare workers treating patients with chronic conditions,<br>• the need the United Nations and other members of international community to press to stop the conflict and access the right to access healthcare of the million besieged civilians in Tigray

Burki, 2022	Commentary	General	• Health workers have not been paid for a year.<br>• Humanitarian workers have been targeted in Tigray including UN and MSF staff<br>• Childhood vaccination rates, which stood at 73% before the war, are now 27%; Coverage with the first dose of the measles vaccine has dropped from 83% to 28%.<br>• An outbreak is a distinct possibility as nearly 900,000 children younger than 5 years have missed the polio vaccine<br>• Half the population in Tigray may now lack access to clean water; and Cholera kits are in short supply.<br>• The 312 ambulances that served the health-care system have been reduced to 38.<br>• Most of the 43,140 Tigrayan patients with HIV who were prescribed antiretroviral therapy have been lost to follow-up, as have patients with tuberculosis.<br>• Covid-19 care system collapsed with only less than one in 10 people got the vaccination and no oxygen for critically ill patients.<br>• Essential medicines are scarce where Tigrayans are in need of vaccines; treatments for HIV, tuberculosis, and malaria; severe acute malnutrition kits; and reproductive health kits.<br>• Scabies is being treated with expired permethrin<br>• 240 patients, including a teenager with HIV and a baby with meningitis, were sent home after the hospital ran out of food.<br>• The global acute malnutrition rate stood at 28%, far higher than the Emergency where 10% is the threshold.<br>• Telephone lines were cut in June, 2021<br>• The federal government in Addis Ababa imposed a punishing blockade.<br>• Barely any fuel has been permitted to enter, severely curtailing the prospects for travel, especially outside the state capital, Mekelle; and Banking services have been suspended, making it extremely hard to get hold of cash, and access to electricity is patchy or non-existent.<br>• The vast majority of the 7 million people who live in Tigray are struggling to find enough food.<br>• Almost half a million children are thought to be malnourished<br>• There have been widespread reports of forced displacement, sexual violence, and mass killings, as well as serious violations of international humanitarian law<br>• accusing forces from the Amhara region in western Tigray of ethnic cleansing and crimes against humanity.<br>• The multiple bureaucratic restrictions on things like visasâ€ by the Ethiopian government to including international aid works and suspension of some NGOs including MSF and the Norwegian Refugee Council not to deliver services in Tigray<br>• Along with ending the effective siege, it is critical that all services, including banking, are restored as itâ€™s impossible deliver healthcare or humanitarian aid on any kind of scale without electricity, fuel, or cash.

Clarfield et al, 2022	Commentary	General	• Ayder hospital has been forced to cancel basic surgeries<br>• In Ayder Hospital, which only recently could boast of its achievements in providing haemodialysis to those in need, patients with renal failure are dying before the eyes of attending staff due to the lack of basic supplies<br>• The availability of essential medications has, as estimated by the Ayder Hospital, plummeted from almost 80% 1 year ago to less than 20%, and laboratory tests have dropped from 94% to less than 50%. Patients are dying from a simple lack of a reliable oxygen supply.<br>• Due to the blockade of Tigray, spare parts for all medical machinery are not just in short supply-- they are non-existent.<br>• Neurosurgeons are operating without the benefit of imaging, depending on clinical skills only a situation reminiscent of the 19th century.<br>• a medical catastrophe is unfolding, in the background of war, famine, and a humanitarian tragedy.<br>• Hospital staff have gone unpaid for most of 2021; and for those who still have some savings, the banks are closed. It is becoming impossible to feed the staff and their children<br>• The authors demand that the relevant bodies, the UN, the African Union, WHO and, above all, the Ethiopian Government stand by the health workers and the patients in Mekele.
Cousins S, 2022	Commentary	Women with fistula	• more than 80% of health facilities have been destroyed<br>• there had been a drastic increase in the number of obstetric fistula cases, although difficult to know the magnitude. In addition to lack of medicines, and the collapse of transport, a major contributor to the increase of fistula was widespread rape.<br>• The conflict has killed tens of thousands of people and displaced millions, while countless women have suffered rape and sexual assault.<br>• Women with fistula require sophisticated counselling and specialised surgical care units to avoid a life of social rejection and isolation but none of these services exist at this point and going to continue to be a huge concern
Devi S, 2022	Commentary	General	• Published in November 2021, only 40 of 224 health centres in Tigray were functional<br>• In Tigray, single-use items such as gloves, surgical materials, and even chest drains are being washed and reused, increasing the risk of infections; and in some places, doctors have replaced disinfectant by salt to clean wounds.<br>• The systematic blockade in Tigray left with no medicine whereby people are dying, no food whereby people are starving, no telecommunication whereby they are isolated from the rest of the world, and no fuel and no cash.<br>• The International Committee of the Red Cross (ICRC) resumed cargo flights on Jan 26, 2022, delivering supplies, including insulin, oxytocin, tetanus toxoid, gloves, and surgical material.<br>• WHO began delivering 33.5 tonnes of health supplies and equipment to Tigray on Feb 11, 2022, the first the agency has been permitted to deliver since July, 2021.<br>• In Tigray, 13% of children younger than 5 years and half of pregnant and breastfeeding women are malnourished

Devi S, 2021	Commentary	General	• Barely one in ten health facilities were functioning in Tigray and many were deliberately looted, vandalised, and destroyed in April 2021.<br>• MSF members, with their MSF-marked vehicle, were stopped by an Ethiopian military convoy<br>• Thousands killed, men forced to rape family members, and destruction of the health system are among the atrocities being uncovered in Tigray by aid agencies after the Ethiopian Government started a military offensive against the ruling faction in the region last November.<br>• US secretary of state (Antony Blinken), used the term â€œethnic cleansingâ€ for the first time on March 10, 2021, at a foreign affairs committee meeting. He said Ethiopian and Eritrean troops must leave and be replaced by â€œa force that will not abuse the human rights of the people of Tigray or commit acts of ethnic cleansing, which weâ€™ve seen in Western Tigrayâ€.<br>• Almost 2000 people were killed in more than 150 massacres by soldiers, paramilitaries, and insurgents, according to reports compiled by a team from the University of Ghent that crosschecked testimony with family, friends, and media.<br>• MSF staff members also witnessed the extrajudicial killing of at least four men.<br>• On March 25, 2021, more than 500 rape cases had been reported to clinics in Tigray and the total of such cases was likely to be much higher.<br>• Women say they have been raped by armed actors; they also told stories of gang rape, rape in front of family members and men being forced to rape their own family members under the threat of violence. But given the fact that most health facilities are not functioning and also the stigma associated with rape, it is projected that actual numbers are much higher.<br>• Patients with chronic diseases such as diabetes, hypertension, and HIV infection, as well as patients in need of psychiatric care, were without life-saving drugs.
Devi S, 2021	Commentary	General	• The airstrikes in Mekelle, Capital city of Tigray, threatened the safety of UN staff who were working to help civilians in humanitarian need in Tigray.<br>• Nearly half of the pregnant and lactating women in Tigray are also now acutely malnourished.<br>• Medicines and equipment are in short supply in Tigray, where cases of malaria, dysentery, and rabies are Rising.<br>• Eighteen (18) people, including one child, died because of a lack of haemodialysis catheters at Ayder hospital in Mekelle.<br>• Ethiopia has suspended for 3 months the work of two aid groups, MÃ©decins Sans FrontiÃ¨res (MSF) and the Norwegian Refugee Council (NRC), who have been delivering service in conflict Zone in Tigray.<br>• Killings, looting, and the destruction of health centres and farms had triggered a surge in humanitarian need in Tigray.<br>• Nearly 200,000 children had gone without critical vaccinations, and the lack of food had made people more susceptible to deadly diseases.

Devi S, 2021	Commentary	General	• There is an imminent risk of famine in the Tigray region of northern Ethiopia, with some humanitarian agencies prevented from to providing aid.<br>• More than 350,000 people are already living with severe food insecurity in Tigray, where 75% of medical facilities remain destroyed or damaged by conflict.<br>• About 5.5 million people of roughly 6 million in the region now face acute food insecurity.<br>• Declaring a famine would be a politically charged move and famine is not a category in the UN-backed Integrated Food Security Phase Classification (IPC) analysis. The UN estimates that 1.8 million people are in phase 4 or have â€œemergencyâ€ levels of acute food insecurity and 353,000 people are in phase 5 or â€œcatastropheâ€.<br>• Food and aid are hampered by ongoing conflict, the displacement of around 1.7 million people in northern Ethiopia, road blocks and military restrictions, and disruption to last yearâ€™s crop harvesting and this yearâ€™s planting season, said the IPC.<br>• Mark Lowcock, UN emergency relief coordinator, told Reuters that Eritrean soldiers and local fighters were deliberately blocking supplies to the more than 1 million people in areas outside Government control and food is definitely being used as a weapon of war.<br>• WHO said in its latest bulletin that only 74 of 264 facilities were functional, malaria was expected to rise in the upcoming rainy season, and the lack of routine childhood vaccination meant an increased risk of measles outbreak.<br>• The massive displacement of people meant it was difficult to assess the number of people in need in rural areas.
Devi S, 2020	Commentary	General	• local hospitals and health facilities were running low on supplies as they treated wounded people, of whom 80% had trauma injuries<br>• The hospital is running dangerously low on sutures, antibiotics, anticoagulants, painkillers, and even gloves<br>• The hospital was lacking body bags and food supplies were also low, affecting those recovering from surgery. MSF reported that there were doing 120 consultations on a daily average in our clinic and at the end of the day there would still be patients not seen<br>• staff at the hospital had to suspend many other medical services to treat wounded patients<br>• After the conflict started, hundreds of foreign aid workers fled and phone and internet connections in Tigray were shut down.<br>• Tigray region has been under a travel and communications ban by the federal Ethiopian Government.<br>• Aid trucks have been blocked for weeks and humanitarian access at least to federal government-controlled areas would allow delivery of much-needed aid, including food and medical supplies. Approximately 600,000 people were dependent on food aid in the region even before the conflict.<br>• Food had run out for almost 100,000 refugees from Eritrea who live in three camps in Tigray.

Favara et al, 2022	Cross-sectional study	Young people	• Allegations of war crimes, mass destruction and looting including of health care facilities have been reported.<br>• At the time of the second phone call, 2 in 5 young people self-reported at least mild depression and/or mild anxiety, and 16% (95% CI, 9.73-23.06) self-reported both.<br>• Rates of self-reported anxiety were three times higher in November/January than in August/October, and rates of self-reported depression went up by ten percentage points.<br>• For those interviewed after the outbreak, rates of at least mild anxiety were eleven times higher than pre-conflict from 35% (95% CI, 23.44-47.33) to 3% (95% CI, 1.24-7.39).<br>• Rates of at least mild depression more than doubled from 12% (95% CI, 12.31; 4.1-20.51) to 28% (95% CI, 16.52-38.87).<br>• Males reported greater increases in anxiety, females in depression.
Gebregziabher et al, 2022	Commentary	General	• The health system in the Tigray region of Ethiopia has been destroyed.<br>• Only 40 of 268 pre-war ambulances remain<br>• More than 80% of hospitals and health facilities in Tigray have been looted and destroyed<br>• A total of 2000 healthcare workers were reportedly registered in internally displaced people camps in the capital city, Mekelle, as of May 2021.<br>• After the onset of the war, more than 50% of members of the regional health work force were unable to report to their working institutions.<br>• The status of the community health extension workers who were in charge of the health extension program became unknown, with their salaries completely cut-off, and their safety not guaranteed.<br>• An estimated at least 20,000 women and young girls have been raped by Eritrean and Ethiopian forces.<br>• Almost 40% of Tigrayans are suffering from an extreme lack of food, after 15 months of conflict.<br>• The Government deliberately blocked humanitarian aid that aimed to feed and provide life-saving services to the millions affected.

Gebregziabher et al, 2022	Cross sectional study	General	• over 67.7% of health centres (153 out of 226) and over 56% of primary hospitals (13 out of 24) in Tigray were non-functional or not communicating.<br>• Only 25% of all primary hospitals, 10% of all health centres and 0% of all health posts at this point in time were still fully functional<br>• Overall, less than 4% of all healthcare facilities across Tigray were FF as of June 2021<br>• In central Tigray, two out of six health facilities in Tigray are partially functional and the remainder are non-functional.<br>• In the East Tigray zone, only 10% of the health centres (4 of 39) were confirmed to be fully functional, and only one of the five primary hospitals was fully functional. Both of the general hospitals in Eastern Tigray remained fully functional.<br>• Mekelle has fared relatively well with 82% of the health centres, one of the two general hospitals, and the zoneâ€™s only referral hospital confirmed remaining fully functional. However, note also that only one of the two primary hospitals in Mekelle was functional.<br>• In the North West Tigray region, only 15% of health centres (6 of 40) were fully functional or partially functional, only one of the two general hospitals remained confirmable as fully functional, and only one of the four primary hospitals remained fully functional.<br>• In the South East Tigray region, less than half of health centres (9 of 22 partially functional and 0 of 22 fully functional) and only one of the three primary hospitals were confirmable as fully functional or partially functional.<br>• In the South East Tigray zone, there were no general hospitals (a situation that existed prior to the onset of this war). Two of the three primary hospitals were confirmed non-functional or not communicating and none of the regionâ€™s 22 health centres was fully functional (only nine are partially functional). In this region, only 4% (1 of 25) of all healthcare facilities remained fully functional.<br>• In the South Tigray region, over one-third of health centres (12 out of 32) were confirmed as fully functional or partially functional, all of general hospitals were confirmed to be fully functional or partially functional, and all primary hospitals were fully functional. In the West Tigray region, less than 5% of health centres (1 out of 21) were fully functional or partially functional, and almost no health facilities were communicating; that is, over 95% of these facilities were under occupation by Eritrean or Amhara forces.<br>• The authors recommend global health associations to list war as a priority health issue.
Gesesew et al, 2022	Commentary	Women & girls	• 250 war-victim women and girls had been admitted to the clinic due to fistula<br>• Fistula patients could not get treated as they cannot afford cost of transportation and rehabilitation<br>• Risk factors to fistula among women and girls included obstructed labor, malnutrition, gang-rape, & sexually transmitted infections.<br>• 36/40 hospitals, 208/232 health centers, and 670/741 health posts had been fully destroyed or partially damaged and 274/308 ambulances had been non-functional i.e. burned or looted.<br>• ANC & skilled delivery decreased from 94% to 16% and 81% to 21% in 2019 to 2022 respectively, leading to obstructed labor during birth<br>• There has been an imposed medical siege in the region since June 2021 and the referral system and communication is broken.<br>• 79% of pregnant & lactating mothers were acutely malnourished in Tigray in October 2021, and 90%+ of the population lack regular access to food<br>• Fistula incidence in Tigray conflict zone is now equally high in women from urban and educated backgrounds as it was in the rural, uneducated, and poor women before the start of the conflict.<br>• the weaponization of sexual violence in Tigray has been documented in many reports, including by Amnesty international.

			• Tigray regional health bureau reported that 7.5% of those women and girls who reported that they were raped were diagnosed with an sexually transmitted infections, and of which 5% were HIV-positive.
Gesesew et al, 2021	Analysis	General	• After the war, virtually the entire MCH service was collapsed and none of the services above were available.<br>• After the onset of the war, a report by the Health Bureau of the Interim Tigray Administration10 revealed that the HEP has become completely non-functional.<br>• Two-thirds of the Woreda Health Offices in the region became non-functional, while the status of the remaining one third (south Tigray, southeast, western and some part of eastern Zones) is unknown as they were under the control of the invading Eritrean and/or Amhara forces.<br>• Out of the assessed 40 hospitals, 21 were non-functional, 12 were partially functional although with severe limitations and the status of the remaining 7 was unknown as they were still under the occupation of the invading Amhara forces and militia.<br>• Out of the assessed 228 health centres in the region, only 40 (17.5%) were functional, while the remaining were either completely non-functional, partially non-functional or their status was unknown due to occupation by the invading Amhara or Eritrean forces.<br>• A total of 238 (90%) of the 269 ambulances which have been serving before the onset of the war were either destroyed or looted.<br>• The intentional damage of healthcare facilities in Tigray as a potential weapon of war with deliberate destruction and looting has led to displacement of thousands of healthcare workers including death of more than ten workers.<br>• A total of 2000 healthcare workers were reportedly registered in internally displaced people camps in the capital city, Mekelle, as of May 2021.<br>• After the onset of the war, more than 50% of members of the regional health work force were unable to report to their working institutions.<br>• The status of the community health extension workers who were in charge of the health extension program became unknown, with their salaries completely cut-off, and their safety not guaranteed.<br>• UNICEF revealed that the level of severe acute malnutrition (SAM) in Tigray in January 2021 was three times of the global WHO emergency threshold, putting 70000 children at risk of death.<br>• MSF found that 26.6% of 309 children screened in mobile clinics at several locations in Northwest Tigray were malnourished and 6% of them were severely acutely malnourished, leading them to conclude this â€˜warrants immediate actionâ€™.<br>• Although there is no reported outbreak to date, given the collapse of the healthcare system, interruption of childhood vaccination, poor sanitation and massive internal displacement, common outbreaks such as cholera, measles, malaria, yellow-fever and COVID-19 are likely to arise or worsen.<br>• There are reports from the WHO which indicate signs of acute watery diarrhoea cases in different towns of Tigray.<br>• There are also many anecdotal reports of patients with insulin dependent diabetes dying after they run out of their insulin supplies.<br>• The United States Agency for International Development (USAID) report found that 39% of women and girls reported being raped inside their homes, and 33% of the victims were gang-raped. Moreover, 44% and 33% of the victims were raped by Ethiopian and Eritrean soldiers respectively, 11% did not know who raped them, and 6% by Amhara Militia, and 6% by combinations of Ethiopian and Eritrean soldiers.<br>• Amnesty international revealed that health facilities in Tigray recorded 1288 cases of gender-based violence between February and April 2021. Adigrat Hospital alone recorded 376 rape cases from the beginning of the conflict to the 9 June 2021.<br>• The World Food Programme reported that 5.2 million people (91% of the total population in the region) are food insecure requiring immediate humanitarian assistance, of which 1.2million people women and children will be assisted with nutritionally fortified food by WFP.

			• MSFâ€™s mobile clinics were implemented as an alternative response, until suspended by the Federal government; Norwegian Refugee Council (NRC) used to support refugees refugee camps for 33,000 Eritreans in Tigray until the federal government suspended it.<br>• Other humanitarian organisation such as the UNAID and other UN agencies were responding to crisis by refilling medications and providing therapeutic food for malnutrition.<br>• Massive displacement of people and lack of access to insecure areas are just a few consequences of the war that further hamper objective assessment of the situation.
Hadera et al, 2022	Commentary	General	• Stressed health workers due to constant attack of the drones<br>• No salary and access to transport to go to work for health workers<br>• Lack of enough medications, supplies and emergency services to patients including oxygen and basic test kits.<br>• 1425 civilians have been the victims of indiscriminate airstrikes (residential areas, hospitals, markets, public transport and other civilian infrastructure<br>• Pregnant and lactating mothers and other women, constitute45% of the total dead and injured.<br>• Blockade of NGOs who provide health services<br>• spike in death rates<br>• drop in the quality of healthcare services at hospitals<br>• deaths of more than 183 neonates in the past six months and 31% of patients admitted to the ICU.<br>• Cancelation of 3700 and above surgeries due to a lack of oxygen in 12 months.<br>• Mortality rate at the emergency department increased from the 0.3% pre-war level to 2.5% at a regional level.<br>• unsafe blood transfusion as blood banks have run out of testing kits to check for HIV, hepatitis, syphilis
Hailu et al, 2022	Cross sectional study	hypertension patients in health facilities	• Follow-up/visit of hypertension patients sharply decreased by 85%, 59%, 85%, 100%, and 11% in Eastern, Northwestern, Southeastern, and Southern zones but increased by 59% in Mekelle after the war.<br>• The study did not include western zones due to occupation and ongoing war, and primary hospitals from central and south zones due to destruction of health facilities data.<br>• The study didnâ€™t include private health institutions and referral hospitals in Axum and Mekelle due to unavailability of data.
Hiluf et al, 2022	Letter to Ethiopian Ministry of Health	Cancer patients	• near total collapse of Tigray health care system<br>• Displacement, death, and destruction as normal elements in war torn Tigray and putting 9 million people inn untold misery.<br>• lack of money, transportation, and communication barriers resulted from the de facto humanitarian blockade<br>• significant loss of therapeutic and survival benefits of cancer patients in Ayder hospital due to scarcity of supplies and chemotherapeutic agents, remaining only 4 chemo drugs<br>• lack of reagents e.g. serum electrolytes to run work up for cancer patients; lack of IV fluid, antibiotics<br>• Not functioning of CT Scan, MRI and mamography<br>• *Physicians facing (and frustrated) painful decisions of sending patents home to die, or sometimes using expired chemotherapeutic drugs.<br>• 87% of health facilities have been looted, vandalized, and destroyed in a deliberate and widespread attack on health care.

			• 1643 adults and 126 children with cancer visited Ayder<br>• The lives of 1769 cancer patients at risk of death or are most likely already dying at home or elsewhere being unable to get proper cancer care in Ayder.<br>• Terminal cancer patients suffering from pain due to lack of potent analgesics e.g. morphine<br>• Health workers call<br>beseech global oncology association to raise their voices re the collapsed cancer care in Ayder<br>• the Federal Ministry of Health of Ethiopia to support essential medical supplies for cancer patients in the besieged Tigray<br>• International community to press to stop the conflict
Hiluf et al, 2022	Commentary	Cancer patients	• a complete communication blackout, with absence of telecommunication, internet, electricity, and other basic services such as banking and transportation.<br>• a medieval-type siege remains imposed on Tigray by the Ethiopian government and its allies.<br>• restriction of food provisions and medicines.<br>• lack of medical supply for cancer patients<br>• treating terminally ill people with expired medication and paracetamol [acetaminophen] in Ayder referral hospital<br>• health workers crying—traumatized vicariously<br>• Health workers urging to raise awareness of the humanitarian catastrophe in Tigray today. No human being should be made to suffer like this<br>• International community attention to Ukraine leaving the worst Tigray crisis
Hiluf K, 2022	Letter to Ethiopian Diabetic Association	Diabetes patients	• There are 26,768 people living with diabetes (including 8,224) and many new diagnosis coming from all over Tigray with advanced form (DKA). However, Ayder hospital is without single vial of insulin nor oral hypoglycemic medications, no dialysis service nor a supply to undergo corrective amputations for diabetic patients in need.<br>• There are 12 diabetic patients dying every week<br>• Ayder staff plead Ethiopia diabetic Association to send diabetic health care supplies and IV fluids for DKA management by any means including through airlifting.
HRW, 2021	Report	women and girls; health/aid workers; refugee members	• only 29 of the nearly 230 health centres in Tigray were fully functional as of April 2021.<br>• Only 1% of health facilities in Tigray had the capacity to provide comprehensive gender-based violence services in April 2021.<br>• The presence of Ethiopian, Eritrean, and Amhara forces with in communities in Tigray in the first half of 2021 also impacted the work of mobile clinics and community outreach programs.<br>• There were reports of at least five instances in which armed groups threatened, harassed, or detained Tigrayan aid worker s supporting sexual violence survivors or providing reproductive health services for local organizations or international organizations.<br>• Health workers and humanitarian aid providers in Tigray described threats and intimidation linked to their work on sexual violence, and their fear of reprisals from Ethiopian authorities.

			• The health workers also said that the presence of armed forces and groups in communities, including Ethiopian, Eritrean, and Amhara forces, impeded their ability to protect privacy and confidentiality, and to run mobile clinics and community outreach programs.<br>• Aid workers said that without clear, consistent access, they Struggled to adhere to best practices for supporting survivors of gender-based violence, including to provide private, women-friendly spaces.<br>• Mental health care and psychosocial support services, including community-based support and specialized services, are massive gaps in Tigray, as well as other conflict-affected regions<br>• Sexual violence survivors sought the following services: testing, treatment, and termination of pregnancy, testing and treatment for sexually transmitted infections including HIV and Hepatitis B, and physical trauma, including broken bones, bruises, stab wounds, traumatic fistula, and for depression, anxiety, post-traumatic stress, and other mental health conditions.<br>• The presence of soldiers at checkpoints on the roads of Tigray and near or inside health facilities also deterred survivors from seeking health services.<br>• Rape has been used as a weapon of war, particularly by Ethiopian, Eritrean, and Amhara forces against Tigrayan women and girls.<br>• Ethiopian Ministry of Health reported 1,324 visits to hospitals in Tigray by survivors of sexual and gender-based violence, including in the cities and towns of Mekelle, Adigrat, Axum, Adwa, Shire, and Maichew; where as Tigray regional Health Bureau reported 2,204 survivors sought services for sexual violence at health facilities from November 2020 to June 2021. The actual number far exceeds due to insecurity, deeply rooted social stigma, and the lack of functioning healthcare facilities.<br>• The Ethiopian and Eritrean armed forces, and regional Amhara militias allied to the Ethiopian army committed numerous grave abuses, including massacres, rape between ages of 6 to 80 years old often accompanied with degrading and ethnic-based slurs, and other sexual violence against women and girls; attacks on refugee camps; and destruction of crops and civilian infrastructure, including healthcare facilities and schools in Tigray.<br>• The armed conflict has also been characterized by the Ethiopian government obstruction of humanitarian assistance in Tigray, which the United Nations has characterized as a de facto blockade.<br>• The Ethiopian government is unlawfully restricting and denying desperately needed food, medical supplies and fuel to the population of Tigray.<br>• The Ethiopian government has imposed unpredictable bureaucratic obstacles for aid agencies to obtain visas and permissions for goods; engaged in harassment, attacks, and expulsion of humanitarian workers; and shut down basic services in the region, including banking, electricity, and telecommunications.<br>• The Ethiopian government has effectively besieged Tigray since Tigrayan forces retook control of most of the region in late June 2021; this raises concerns that the Ethiopian government is using starvation as a weapon of war, a violation of the Ethiopian criminal code and a war crime under international humanitarian law.<br>• The lack of safety, information, health facilities, medications, trained staff, and transport prevented survivors of sexual violence from seeking or receiving time-sensitive treatments, including during the critical 72-hour window to administer post-exposure prophylaxis to prevent HIV (more than 80% of survivors did not come within 72 hours) and the 120-hour window for emergency contraception to prevent pregnancy.<br>• Healthcare providers in Tigray need mental health support, as these Tigrayan health workers have done so in the context of having their own lives and communities disrupted by conflict, displacement, and loss, and the shutdown of essential services.<br>• Enforce zero tolerance for sexual violence & other violations of international human rights law & international humanitarian law<br>• Allow immediately rapid, full, safe, and unfettered access to humanitarian aid

			• Strengthen and expand a comprehensive approach to preventing and responding to gender-based violence across Tigray and conflict-affected areas, including at the regional, woreda (district), and village levels.<br>• UN member countries should increase pressure on parties to the conflict to allow rapid and unfettered access to humanitarian aid, and should establish and support an independent international investigation into human rights abuses and war crimes in the Tigray conflict.
Kumar et al, 2022	Commentary	General	• Between mid-December 2020 and March 2021, MSF teams in Tigray assessed 106 health facilities and found that only 13% functioned normally. Health centres were looted (70%), damaged (30%), occupied by soldiers (20%), and/or rendered completely non-functional (65%). In addition, only 11% of ambulances were available for use, with the majority damaged, looted, or seized by armed groups.<br>• The only oxygen-producing company in Tigray no longer functions owing to lack of spare parts and maintenance, jeopardizing care for COVID-19, which was already hampered by the interruption of vaccinations<br>• The pervasive lack of medication endangers the estimated 180,000 patients with chronic disease in Tigray<br>â€¢ The supply of essential drugs at Ayder hospital dwindled to 17.5%, and an estimated 117 patients have died owing to supply shortages.<br>• In addition to the damage inflicted on Tigrays health infrastructure, many healthcare workers have been killed, displaced, or remain unaccounted for.<br>• The lack of safety for health workers caused thousands of them to flee, leading to further health worker attrition. The destruction of health institutions and lack of compensation for healthcare workers has prevented providers from returning to work.<br>• Half of all pregnant and breastfeeding women and 13% of Tigrayan children under 5 years old were found to be malnourished; whereby acute malnutrition increases the risk of poor pregnancy outcomes and can cause developmental delays in children.<br>• Amnesty International has reported that Ethiopian and Eritrean soldiers have used gang rape and the intentional spread of HIV as weapons of war.<br>• Health centers in Tigray reported 1,288 cases of sexual violence between February and April 2021. In the early days of the war, healthcare workers provided care to survivors of sexual violence for termination of pregnancy, testing and treatment for sexually transmitted infections including HIV, and physical trauma.<br>• 21 months of conflict have left thousands dead and millions vulnerable to disease and hunger<br>• The total communications blackout in Tigray has exacerbated difficulties in documenting human rights abuses and prevented the population from accessing health-related information during the COVID-19 pandemic<br>• Approximately 2.1 million people have been reportedly displaced, which has strained resources in a region already confronting food insecurity. In early 2022, 83% of people in Tigray are food-insecure with almost 40% suffering an extreme lack of food.<br>• Comprehensive physical and mental health care is needed to address these humanitarian needs.<br>• The global health community should provide policy expertise and funding to rebuild the healthcare infrastructure in Tigray.<br>• Physicians have resorted to recycling single-use devices such as gloves, chest drains, and surgical supplies, which have led to complications such as infections, kidney failure, and amputations.<br>• Local businesses and community members have donated soap, linen, and bedsheets to sustain hospital operations but damaged imaging equipment remains in disrepair because replacement parts cannot pass through the blockaded.

			• The global health response in Tigray should prioritize the safety and security of health professionals. International aid organizations can rehabilitate the health workforce by training individuals in the Health Extension Program.<br>• Training the healthcare workforce could be implemented virtually by global institutions, and Multi-Donor Trust Funds from the United Nations aid organizations can also provide health financing and assistance for healthcare workers and staff in Tigray.<br>• The global health community should collectively engage the Ethiopian government to demand unfettered access for humanitarian supplies to Tigray.<br>• Humanitarian access could be stepwise: first, access to food, medicines, oxygen tanks, and health professionals; second, the resumption of basic medical services including preventive, diagnostic, and therapeutic procedures; and third, the rebuilding of health centers, and the establishment of new medical facilities, based on current needs.<br>• Global health academics and international aid organizations must advocate on a global stage for full compliance with international law and the lifting of all humanitarian blockades by the Ethiopian government.
Makoni M, 2022	Commentary	Cancer patients	• None of the health facilities in the region, once one of the communities with the best health-care systems in Ethiopia, are currently operating at pre-war levels, as a consequence of the unavailability of power, water, and essential devices, which have been looted or destroyed.<br>• The 19-month-long ongoing war in the northern regional state of Tigray has shattered health provision by destroying health facilities, virtually wiping out care for people with cancer in the region.<br>• Patients with terminal disease were being treated with expired medication and paracetamol before an 11-month government blockade and communication blackout in Tigray were eased in early May, 2022, with an airlift by the International Committee of the Red Cross.<br>• Aid agencies are reported to require permission from the Ethiopian Government to deliver cancer drugs.<br>• The 19-month-long ongoing war in the northern regional state of Tigray has shattered health provision by displacing health workers, virtually wiping out care for people with cancer in the region.<br>• In a humanitarian report in November, 2021, the UN Office for the Coordination of Humanitarian Affairs reported that the cancer treatment programme at the Ayder Referral Hospital in Tigray had closed, leaving some 500 patients without treatment.

Mulugeta et al, 2022	Commentary	Children	• The study found that 199 (6%) of children aged 6-59 months had severe acute malnutrition (MUAC <115 mm), 713 (22%) had moderate acute malnutrition (MUAC â‰¥115 mm and <125 mm), and 912 (28%) had moderate or severe acute malnutrition (MUAC <125 mm; commonly labelled as global acute malnutrition).<br>• Severe acute malnutrition increased significantly from 1% in 2019 (pre-war) to 6%, and moderate acute malnutrition increased significantly from 8% to 22% (both p<0.0001).<br>• Similarly, global acute malnutrition nearly tripled from 10% in 2019 to 28%, which is a staggering and sharp increase.<br>• Household food security dropped significantly (p<0.0001) from 59% in 2019 to 15%, and household food insecurity (mild, moderate, or severe) increased from 41% to 85%.<br>• The sharp increase in the prevalence of acute malnutrition and household food insecurity is a direct consequence of the humanitarian aid blockade and the destruction of food sources stemming from the civil war<br>• Some of the defining characteristics of this armed conflict are a total blockade of humanitarian aid, wanton destruction of health facilities, and the displacement of millions of people
Paltiel O et al, 2022	Commentary	General	• Doctors in Tigray are unpaid and malnourished, doing the unimaginable such as reusing dialyzers, prescribing outdated drugs in inadequate dosages, performing complex surgery without imaging, and all too frequently, sending patients home to die from easily treatable ailments.<br>• In Tigray, child malnutrition is at triple the rate seen in the recent past due to the widespread civil war.<br>• Malnutrition is occurring even in the middle classes who cannot access their savings.<br>• Patients, who one year ago could receive up-to-date care with dialysis or chemotherapy, are dying because of a lack of consumables, drugs, and supplies.<br>• UN estimates that more than 90% of the population in the region is in urgent need of humanitarian assistance.<br>• It is time to support and show solidarity with the dedicated healthcare professionals in Ethiopia who are working around the clock, with extremely limited resources, to provide healthcare to the population. What is missing is food, equipment, and medicine.<br>• World leaders and politicians must step up, and ensure that these resources reach health facilities like Ayder, so that the people of Tigray receive the care they need and deserve, and that their skilled workforce is able to provide, despite their own hunger.<br>•

Phillips et al, 2022	Personal field observation and authors experience	General	• According to the MSF assessment of 106 medical facilities between mid-December 2020 and early March 2021 in the Tigray region<br>• Health extension program (HEP) could provide a platform for the rapid development approaches:<br>• Populations comprised of forced migrants require mass distribution of uncooked commodity food by HEP staff.<br>• community-based appraisal operations could be designed to provide continuous information on the epidemiology of nutritional adversity as well as evidence-driven screening and referral.<br>• Community based approach eases evidence-based screening and referral within priority localities could facilitate the targeting of resources on children in greatest need.<br>• The involvement of community mobilization in the screening and referral process could engage local leaders in sustaining screening and referral operations, obviating the need for widespread international team deployment, while enabling external support to be targeted on acute care.<br>• Current operations in Tigray prioritize acute care needs, but if links to a screening and referral operation could be added, this would enhance the life-saving potential of clinical interventions.<br>• Children who have recovered from acute adversity could be monitored and supported with the provision of ready-to-use therapeutic food, employing MUAC as a community implemented monitoring and referral tool.<br>•

Teka et al, 2022	Commentary	women with cancer	• Generally 70% had been looted, and more than 30% had been damaged; just 13% were functioning normally. During this war, health institutions have been particularly targeted throughout the entire region in Tigray.<br>• With the presence of soldiers at checkpoints on the roads and near health facilities, civilians, especially from outside urban areas, have been prevented from getting medical treatment.<br>• As the result of the damage of health facilities and looting of ambulances, laboring mothers are dying at home. Women with cancer are being sent away to die at home.<br>• For the past 17 months, no chemotherapy drugs have been allowed to enter into Tigray.<br>Ethiopia planned establishing five radiation centres and all radiation centres are completed except Ayder hospitalâ€™s radiation centre which remains under construction as its process was caught in the war. The radiation therapy equipment bought for the radiation center remains idle in the hospitalâ€™s premises.<br>• All diagnostic procedures and tests are no longer available. Both emergency and elective surgeries are being cancelled due to power outage and lack of basic surgical supplies including surgical gloves, intravenous fluids, and anesthesia drugs. Chemotherapy drugs are no longer available and cancer patients are being sent home to die.<br>• This war has left hundreds of thousands of dead, millions displaced, and pushed millions into starvation and famine<br>• The Tigray conflict has resulted in widespread reports of sexual violence in areas controlled by Ethiopian and Eritrean federal forces, and regional Amhara militias. Reports of rape, gang rape, sexual slavery, torture, beatings and killings of family members, and degrading, ethnic-based slurs have been widespread<br>• The authors ask the global community to show solidarity to end the suffering of women who are fighting a war with in a war. The African Union, the United Nations, international donors, the international community, cancer societies, and physicians worldwide should press all parties to allow unfettered humanitarian aid access and to lift the siege throughout the Tigray region.
Teka et al, 2022	Commentary	women with cancer	• About 28% of hospitals, 18% of health centers, 11% of ambulances, and none of the 712 health posts remained functional after six months of the war.<br>• The Ethiopian government imposed a siege on the Tigray region, leading blockade of humanitarian aid blockade, lack of transportation fuel, power outages, communications blackouts, and banking services interruption, and depletion all essential drugs and medical supplies.<br>• running out of screening and diagnostics care and chemotherapeutic drugs; stoppage of surgeries.<br>• Cervical cancer screening services have fallen by half from the pre-war period<br>• substantial reduction of surgical care for gynecologic malignant disease (from 108 surgeries to 79 during the war and to 45 during the siege.<br>• lack supplemental oxygen, anesthetic medications, antibiotics, operation theater materials, and fuel to run generators

Tesema et al, 2021	Commentary	General	• An assessment by United Nations Office for the Coordination of Humanitarian Affairs (UNOCHA) and others indicated that about 70% of assessed hospitals and health centres in the region were either partially or fully damaged during the war, most ambulances were either looted or destroyed and all the health posts were rendered non-functional; leaving over 2.5million people without access to essential services including the millions of internally displaced people.<br>• A recent UN report revealed that the woreda level health administration structure in Tigray is non-existent or has limited function. For example, there is no system to monitor service delivery in the region.<br>• The most recent reports of the killings of MÃ©decins Sans FrontiÃ¨res staff in the Tigray region vividly show the risks to health workers in the area.<br>• Evidence also revealed that women and girls were subjected to sexual abuse and gender-based violence in this war.<br>• The war in the Tigray region of Ethiopia, which started in November 2020, has already caused a considerable number of casualties, massive internal displacement and over 70000 refugees in neighbouring Sudan.<br>• The process of restoring and rebuilding Tigrayâ€™s health system should focus on (a) health system adaptation; (b) protecting, reskilling and empowering the health work-force; and (c) coordinating actors and resources towards creating a sustainable and resilient health system.
TRHB, 2022	Report	General	• A report by World Food Program suggests that 91% of the Tigrayâ€™s six million people require immediate emergency humanitarian assistance, and 400,000 people had crossed the â€˜threshold into famineâ€™.<br>• health facilities in Tigray were deliberately targeted and intentionally made dysfunctional â€“ that includes bombing or shelling, and setting to fire (example: Sebeya Health Center (HC) in Eastern Zone; Bora HC in Southern Zone, and Semema Hospital, Edaga Arbi Hospital and Chila HC in central Zone); looting and vandalism of the entire health commodities from medicine and medical equipment to imaging machines and ambulances and systematic destruction to the water and electric systems of the health facilities.<br>• Several health facilities served as military camps for the ENDF and its allies for several months, including Adi Aynom, Mugulat and Alasa health centers; Dr Tsegay (Fatsi) and Fresemaetat (Hawzen) primary hospitals; Adwa and Abie Adi general hospitals, which were not accessible for civilians.<br>• 73% of the assessed 106 health facilities in Sothern Eastern, Central, Western and North-western Tigray have been either damaged or looted, while 65% were completely non-functional (no staff, no means) and 20% had been occupied by armed groups (not accessible for civilians)â€[illegible] between Dec 2020 & March 2021.<br>• In November 2020 to June 2021, only 18% (40 of the 224 health centers) in Tigray were functional, where health facilities were damaged from shelling, looted of medicines and equipment, and left with an absence of medical personnel; leaving the population in most parts of Tigray without access to health care.<br>• Out of 880 assessed health facilities in Tigray (87% of the total 1,011, except those inaccessible areas in western zone and some districts bordering Eritrea) in July 10 - 30, 2021, 695 (or 79%) health facilities have been damaged. In particular, 82.4% of hospitals (28 of the assessed 34), 80.6% of health posts (514 of the assessed 638), and 73.6% of health centers (153 of the assessed 208).

			• There was only slight differences among zones, where most of the health facilities were damaged and/or vandalized either fully or partially from Central (244, 86.8%), Eastern (131, 91.6%), North-west (132, 76.3%), and South-east zone (76, 80%).<br>• The damaged health facilities are characterized by physical destruction to the buildings including water, sanitation, and electric systems â€" in part or in full, and/or looting and vandalism of medicines, vaccines and medical equipment, furniture, or having inadequate health workforce.<br>• Ayder Referral hospital deteriorates its function due to lack of basic drug supplies, medical equipment, and operational problems including fuel, oxygen supply and cash<br>• The deliberate attack on health care in Tigray is against the 1949 Geneva Conventions and the additional Protocols of 1977.<br>• During the fourteen months of Tigray war crisis (since Nov 2020), health workers have been living under a very stressful situation with no medicine at hand to give care for patients, yet with no salary, no means of transportation or without any incentives.<br>• About half of the health workforce in Tigray were either killed, injured, or displaced<br>â€¢ Death of two health workers and 23 aid workers were reported<br>• 1.7 million women aged 15-49 years missed basic health services, 910,000 children aged 6-59 months missed measles, 893,228 children under five missed basic health and nutrition services, and 297,408 mothers and children diagnosed with acute malnutrition.<br>• Although underrated, 276 mothers died during the conflict, compared to 136 at pre-war.<br>• Only 38 of the 312 ambulances are functionalâ€"around 65% of the deliveries were transported to and from health facilities with these ambulances prior to the current war.<br>• most of the lifesaving medicines and medical equipment are totally absent, including oxygen for COVID-9 patients and dialysis services for renal cases at Ayder Referral hospital and almost in all health facilities in Tigray.<br>• Antenatal care declined from 94% to 16, facility based delivery from 81% to 21%, and postnatal care from 73% to 19%.<br>• Acute malnutrition among Pregnant and Lactating Women increased up to 78% in many of the woredas worst affected, Integrated Food Security Phase Classification phase five (IPC-5) or famine level.<br>• A mortality survey collected representative data between July to October 2021 from 333 sub-districts (40% of the total villages) in all zones of Tigray, except from Western Tigray. The survey showed, 5421 deaths (57% males) were recorded and estimated 40,000 deaths in 813 villages. Many of the deaths occurred at home (77%) and across all ages, though higher in the age group 15 years and above. The morality burden has been higher in Central, Eastern and North-west zones.<br>• There was no information about the status of 24,253 diabetic patients on regular treatment in the year 2021, except 1,598 deaths; and no information about the 29,271 hypertensive patients except 2,385 deaths.<br>• There were 43,140 patients on anti-retroviral treatment and 2,363 pregnant mothers taking ARV prophylaxis with 867 HIV exposed infants in Tigray befreo the start of the conflict. In a quick record assessment, 81% of the 381 HIV patents were found lost to follow up in 2021.<br>• From, July to September 2021, 90% of tuberculosis patients have been lost (105 of the 118 sampled) also implying rug resistance-- there were 6,699 newly detected Tuberculosis (TB) cases (All forms) in 2020.<br>• prior to the Tigray-war, there were 6,764 cases (7.7% of the 88,155 tests conducted); however, only 8,832 tests (10% of the pre-war) were conducted after the war, with increased positivity rate to 26% (2,306 cases). The Covid-19 death rate also showed four-folds increment, from 0.7% to 2.8% (47 death out of 6,764 cases in 2020 and 64 death out of 2,306 cases in 2021).

			• The war resulted in the massive displacement of people from all directions towards urban centers such as Shire, Axum, Adwa, Adigrat, Abie Adi, Maychew and Mekelle and towards deep rural villages of Tigray.<br>• More than 1.2 Million Tigrayans in Western Tigray have been forcibly evicted from their homes, currently in a destitute life as IDPs within Tigray, while over 70,000 fled to Sudan.<br>• As of December 2021, more than 5.2 million people, including 2.1 million IDPs faced with a dire need of humanitarian support.<br>• the entire seven million Tigrayans are currently under a total communication blackout, life threatening air and drone strikes, and suffering from lack of basic life support amenities, such as food, shelter, water, medicines, cash, and fuel.<br>• Starting January through June 2021, alternative health care approaches such as through 65 mobile clinics have been provided under a very limited capacity.<br>• COVAX and Oral Cholera vaccine (OCVs) have been started in April and June 2021 aimed at reaching 1.6 Million and 2 million targets respectively with two doses each, although the program was interrupted due to electricity interruption.<br>• Health cluster coordination and partnership were attempted using the existing structures to humanitarian response such Reproductive, maternal, neonatal and child health (RMNCH TWG), Public Health Emergency Management (PHEM TWG), Risk Communication and Community Engagement (RCCE) taskforce, COVID-19 Taskforce and Pharmacy, Logistics and supplies.<br>• Although a total budget of USD $1.3 Billion was requested for Tigray response, including the $48M from WHO for the period of July to December 2021 for health sector response, the promised budget has not been disbursed due to blockage, closure of banking services, and other reasons.<br>• Prepare working plan for 2022, with detail supplies and cash requirements, as a cluster.<br>• Issue weekly advocacy and call for ACTION updates (unfettered access to medicine, vaccines, and medical equipment) substantiated with concrete evidences on the ground, including the horrible stories of people dying from lack of humanitarian support.<br>• accountability â€“ weekly and monthly updates on what is available (cash and supplies) and what is required immediately to save the lives of seven million Tigrayans.<br>• Strengthen the cluster coordination at Tigray level, as well as at Zonal level through physical presence of international professionals on the groundâ€"this applies to the Health Cluster (WHO), Nutrition & WASH Clusters (UNICEF) who are directly impacting the health outcome<br>• Join hands with other clusters and humanitarian actors to end the de-facto blockade, air and drone strikes, and solve some of the pressing challenges such as fuel, cash, and communication

TRHB, 2022	Letter to GAVI	Children requiring vaccination	• collapsed health infrastructure<br>• children denied access to vaccination, leading to major outbreak; and the electricity interruption lead to discarding the available stored vaccines leading to stock out of vaccines.<br>• vaccine preventable outbreaks are more observed in rural residents due lack of transportation and communication. Measles outbreaks in 10 Districts, reports of 3 Acute Flaccid Paralysis (possibly poliomyelitis), 14 cases of pertussis, and increased Neonatal tetanus (NNT) witnessed in Tigray.<br>• Death of children from vaccine preventable illness<br>• coverage of each vaccine pre- and post war in graph provided, showing the sharp declines of overage of all vaccines.<br>• calling GAVI to to note the man made denial of access to vaccinations, and pressurize the Ethiopian government to deliver vaccines.<br>• vaccine in Tigray for six months since March 2022<br>• imposed a siege/de facto blocked in Tigray by Ethiopian government leading to suffering, harrowing catastrophe, and loss of lives due to shortage of food, medicine and medical supplies
Wall L, 2022	Commentary	General	• Ethiopia, Eritrea and Amhara forces looted and vandalized hospitals, clinics, health Posts in Tigray, and ambulances and carted their booty back to Eritrea in military convoys.<br>• Only a few hospitals in Tigray are functional at any capacity, the transportation network has fallen apart, the telecommunications network has been destroyed, and access to safe, timely cesarean delivery has dwindled to almost nothing.<br>• Health workers and humanitarian aid workers were harassed and prevented from distributing supplies.<br>• Doctors have to go out and beg for food after completing their shifts if they are to feed their families.<br>• Nurses and surgical technicians are fainting during extended operations from lack of nourishment, but the number of operations is rapidly falling because of lack of anesthetic drugs, surgical sutures, intravenous fluids, and the constantly diminishing institutional capacity.<br>• Rape was used as a weapon of war to terrorize the Tigrayan population.<br>• Catastrophic complications of obstructed labor have become common place, because the healthcare infrastructure has collapsed.<br>• The world medical community must speak out and condemn against the horrors that are occurring in Tigray, the use of rape as a weapon of war, the use of starvation to achieve political ends, and the deliberate destruction of health care systems and the intentional harm inflicted upon civilians for revenge and partis an political gain.<br>• Thousands of civilians were killed, often in extrajudicial executions. Tens of thousands of Tigrayans fled across the western border, seeking refuge in Sudan, accompanied by the bodies of their not-so-lucky countrymen, which floated down the Tekeze River near Humera. hundreds of thousands of Tigrayans are threatened by starvation as the result of a deliberate, man-made famine promulgated by the invaders.<br>• Millions more are now internal refugees, flooding into Mekelle and facing acute food insecurity, where the genocidal plan is working.

Weledegebriel et al, 2022	Article	HIV patients	• The study included 33 health facilities from 25 rural districts were to assess HIV service delivery.<br>• clinical follow-up of HIV patients declined from 2409 in October 2020 (pre-war) to 782 in November 2020.<br>• number of HIV patients on ART decreased from 1912 in September 2020 (pre-war) to 318 in January 2021 (war period), a 83.4% decrement.<br>• Trend of laboratory services also declined where 1004 patients (pre-war) tested for laboratory services compared to 149 HIV patients in November 2020.<br>• The number of patients in the sampled health centers reduced from 1123 in September 2020 to 290 in November 2020, and from 1293 pre-war to 492 during the war period in the sampled primary hospitals.<br>• The number of patients on ART in the sampled health centers reduced from 755 in September 2020 to 448 in November 2020, and from 1185 pre-war to 492 during the war period in the sampled primary hospitals.<br>• In the Zones of Tigray, compared to September 2020, the flow of patients with clinical follow-up in November 2020 was 0 of 521 (0%) for North west zone, 426 of 1250 (28.2%) for Central zone, 426 of 1250 (34.1%) for Eastern zone, 113 of 230 (49.1%) for South East zone, and 170 of 201 (84.6%) for South zone.

Weldegerima et al, 2022	Commentary	Cardiac patients	• Nearly 20 months on, displacement, death, and destruction of healthcare facilities have become the defining elements of this armed conflict, putting millions of lives at risk.<br>• The war has led to a near-total collapse of the Tigray regional healthcare system, rendering 70-80% of healthcare facilities in the Tigray region dysfunctional.<br>• Due to the communication blackouts, 16 months into the conflict, the number of Cath Lab procedures and the number of cardiac patients followed in the cardiology unit has dramatically decreased by 50%, leading to the untimely loss of lives with a significant number of preventable home deaths: The number of Cath Lab procedures in 2015 to 2022 is 1, 64, 91, 84, 127, 26, 19, and 7 respectively.<br>• Number of hypertension patients in Ayder hospital reduced from 1800 in 2020 to 900 in February 2022, Rheumatic heart diseases from 1400 to 700, Congenital heart disease from 638 to 319, and ischemic heart disease from 600 to 300, and cardiomyopathies from 400 to 200.<br>• Patients who were receiving at least the basic heart failure drugs are now being sent home by their physicians due to the lack of critical cardioactive drugs and diagnostic tests.<br>• Ayder Hospital has also run out of coagulation profile tests, which are also lacking in Tigray. Computed tomography scan or magneticresonance imaging services are not any longer available.<br>• The steeply deteriorating diagnostic and therapeutic resources are causing a huge psychological impact on patients and practitioners.<br>• Ayder Hospital staff and their partners are urgently speaking out about the disastrous impact that this war is causing on healthcare delivery.<br>• The authors request that the national and international cardiology communities provide an urgent response to restore healthcare, prevent further loss of life, and ameliorate the misery of our patients. They urge and call upon all professionals and organizations to be the advocates of our patients and bring support to the patients in Tigray, Northern Ethiopia
WFP, 2022	Report	general population	• While the overall proxy GAM rates for 6-59 months were estimated at 12.7%, of which proxy SAM rates were 3.6%; the proxy GAM rates for children aged 6-23 months was 25.5% and SAM rates of 10% children but, was 7.5% of GAM and 1% of SAM for 24-59 months children.<br>• Out of 279 pregnant or lactating women among the 979 households assessed, the proxy MAM rates were found to be 60.5%, comparable to the average of 53.3% from more than 250,000 screened through September 2021.<br>• only 15% of the pregnant or lactating women classified as moderately malnourished were reportedly taking any supplements (e.g.,SuperCereal sachets) in the 30 days prior to the survey, potentially underscoring the high caseload of unmet needs for PLW continuing to face inadequate dietary intake and lack of access to essential maternal health care services.<br>• Food security in the Tigray Region is worrisome with 83% of households being food insecure (of which 46.4% are moderately food insecure and 37.5% are severely food insecure). The zones recording the highest food insecurity rates are the North western zone (93%), Eastern zone (86%) and the Central zone (84%).<br>• Across the region, 86% of the assessed households identified themselves as host community, 4% identified that they were currently displaced, 4% identified as returnees and a final 5% identified as being of mixed displacement status.<br>• In November, 52 percent of the assessed population reported planting during the recent Harvest season planting season leading up to the data collection period.<br>• Re household hunger scale, 45% of surveyed households were categorised as experiencing little to no hunger, whereas 40% and 15% of surveyed households were categorised as experiencing moderate and severe hunger respectively.

			• 76% of households were classified as engaging in high or extreme levels of consumption-based coping strategies<br>• The ability to meet the urgent need for increased food supplies through external sources.<br>• Critical monitoring of nutrition, mortality, and health outcomes to monitor the severity of the situation over time and to immediately flag any further deteriorations<br>• Monitoring of household outcomes and contributing factors to acute food insecurity in areas of highest concern, to inform operational partners and to prioritise assistance to communities most in need.<br>• Preparations for this face-to-face survey required careful Planning, primarily related to the lack of communication, limited amount of humanitarian fuel, and limited contingency plans in case of emergencies.<br>• The fact that certain areas were removed leads to geographical/accessibility bias
Yemane et al, 2022	Commentary	women of reproductive age	• Health centres, clinics and hospitals throughout Tigray were systematically targeted for destruction, in contravention of the Geneva Conventions and the internationally accepted laws of war.<br>• As a result of the blockade of food and medical supplies to Tigray, even the most rudimentary services at our hospital have started to vanish, as has been previously reported.<br>• We are forced to wash and re-use examination gloves<br>• In Tigray, anti-D immunoglobulin is no longer offered to Rhesus (Rh)-negative women, pharmacy is depleted, and the few drugs that remain are beyond their official shelf lives; and essential drugs such as misoprostol, intravenous fluids, hepatitis B vaccine, antibiotics, chemotherapy agents, diclofenac, surgical sutures and bandages are now unavailable; and laboratory investigations, such as haemoglobin, urinalysis and rudimentary organ function tests, are no longer available.<br>• Global acute malnutrition among children now affects 28% of the paediatric population, and is worsening under conflict conditions.<br>• There are multiple reports of gang rapes that lasted for days, sometimes ending in the deliberate mutilation of the victims.<br>• Civilians have been massacred, crops destroyed, livestock slaughtered and schools looted<br>• Some 70,000 Tigrayans have f led across the western border to Sudan as refugees.<br>• Two million Tigrayans have become displaced internally, many fleeing to Mekelle.<br>• Nearly 90% of the population of Tigray now live in conditions of food insecurity, and some 400,000 people face starvation.<br>• A de facto humanitarian blockade on the delivery of food and medical supplies to Mekelle was imposed from the beginning, even when the city was under the control of federal troops.<br>• We implore the international community of obstetricians and gynaecologists to raise their voices to demand an end to the blockade of humanitarian aid to Tigray.<br>• The siege must be lifted. The women of Tigray urgently need access to these lifesaving medical supplies.
Gebrearegay et al, 2022	Case study	Kidney cases	• At least 22 humanitarian aid and health workers have been killed; only 1300 of the more than 20,000 health care workers prior to the war in Tigray are reporting to their duties.<br>• Standardized PD solutions and catheters are not always available in developing countries. This challenge leads to the use of improvised equipment in the management of critically ill children with AKI patients, using a modifed nasogastric tube as a catheter and fortifed Ringers lactate as a PD solution.

			• In patients with cardiac impairment, PD is a viable alternative to hemodialysis also because the cardiovascular tolerance of PD is excellent, a challenge leading the clinicians to use of improvised equipment
Berhe et al, 2022	Case study	Kidney patients	• Kidney care provision in the embattled Tigray region has been compromised to an unprecedented extent. Patients with end-stage kidney disease can neither be offered optimal dialysis, nor can they be referred for renal replacement therapy elsewhere.<br>• The authors urgently call upon the international nephrology societies and kidney transplant associations to advocate access to immunosuppressive medications for kidney transplant recipients in Tigray, Northern Ethiopia, to avert additional catastrophic events like the reported one.

List of included studies

1. Berhe E, Paltiel O, Teka H, et al. Ethiopia's Tigray Dialysis Service Cut Due to Dwindling Supplies Amid War. *Kidney International Reports* 2022;7(5):1136-37. doi: 10.1016/j.ekir.2022.02.024
2. Berhe E, Tesfay B, Teka H. Vicarious trauma on the hemodialysis healthcare workers in the besieged Ethiopia's Tigray region: a call to action. *BMC medicine* 2022;20(1):431. doi: 10.1186/s12916-022-02637-1
3. Burki T. Humanitarian crisis in Tigray amidst civil war. *The Lancet Infectious diseases* 2022;22(6):774-75. doi: https://dx.doi.org/10.1016/S1473-3099(22)00304-8
4. Hiluf K, Mustefa M, Irgau I, et al. War-Inflicted Crisis in Cancer Care: The Case of Tigray. Washington: ASCO Connections 2022.
5. Cousins S. "Every challenge is here": fistula in Ethiopia. *Lancet (London, England)* 2022;400(10353):647-48. doi: https://dx.doi.org/10.1016/S0140-6736(22)01634-8
6. Gebregziabher M, DeLargy P, Jumaan A, et al. War prevention and mitigation are public health imperatives of our time. *EClinicalMedicine* 2022;47:101385. doi: 10.1016/j.eclinm.2022.101385 [published Online First: 20220414]
7. Kumar WM, Gebregziabher BA, Mengesha RE, et al. Humanitarian aid must be allowed to enter the besieged Tigray region of Ethiopia. *Nature medicine* 2022;28(9):1734-35. doi: 10.1038/s41591-022-01921-0
8. Wall LL. The Siege of Ayder Hospital: A Cri de Coeur From Tigray, Ethiopia. *Female pelvic med* 2022;28(5):e137-e41. doi: https://dx.doi.org/10.1097/SPV.0000000000001181
9. Makoni M. Conflict in Tigray impeding basic care for patients with cancer. *The Lancet Oncology* 2022;23(7):842. doi: https://dx.doi.org/10.1016/S1470-2045(22)00328-X
10. Paltiel O, Clarfield AM. Tigray: the challenges of providing care in unimaginable conditions. *BMJ (Clinical research ed)* 2022;376:o400. doi: https://dx.doi.org/10.1136/bmj.o400
11. Tesema AG, Kinfu Y. Reorienting and rebuilding the health system in war-torn Tigray, Ethiopia. *BMJ global health* 2021;6(8) doi: https://dx.doi.org/10.1136/bmjgh-2021-007088
12. Teka H, Yemane A, Berhe E. War and Siege Halt Gynecologic Oncology Services for Women in the Tigray Region of Ethiopia: A Call to Action. *JAMA oncology* 2022 doi: 10.1001/jamaoncol.2022.6106
13. Gesesew HA, Berhe KT, Gebretsadik S, et al. Fistula in War-Torn Tigray: A Call to Action. *Int J Environ Res Public Health* 2022;19(23) doi: 10.3390/ijerph192315954 [published Online First: 20221130]
14. Hadera AE. Tigray: Deadly skies, helpless hospitals. *BMJ* 2022;379:o2982. doi: 10.1136/bmj.o2982
15. Yemane A, Teka H, Tesfay F, et al. Obstetrics and gynaecology in an Ethiopian war zone. *BJOG: An International Journal of Obstetrics and Gynaecology* 2022 doi: 10.1111/1471-0528.17238
16. Clarfield AM, Gill G, Leuner CJ, et al. An appeal to world leaders: health care for Ethiopians. *Lancet* 2022 doi: 10.1016/s0140-6736(22)00054-x [published Online First: 2022/01/16]
17. Devi S. Medical aid returns to Tigray. *Lancet (London, England)* 2022;399(10326):707. doi: https://dx.doi.org/10.1016/S0140-6736(22)00309-9
18. Devi S. Aid blocked as Tigray faces catastrophic hunger. *Lancet (London, England)* 2021;397(10293):2451. doi: https://dx.doi.org/10.1016/S0140-6736(21)01433-1
19. Devi S. Further setbacks for Ethiopia humanitarian missions. *Lancet (London, England)* 2021;398(10311):1558. doi: https://dx.doi.org/10.1016/S0140-6736(21)02380-1
20. Devi S. Tigray atrocities compounded by lack of health care. *Lancet (London, England)* 2021;397(10282):1336. doi: https://dx.doi.org/10.1016/S0140-6736(21)00825-4
21. Devi S. Humanitarian access deal for Tigray. *Lancet (London, England)* 2020;396(10266):1871. doi: https://dx.doi.org/10.1016/S0140-6736(20)32669-6
22. Mulugeta A, Gebregziabher M. Saving children from man-made acute malnutrition in Tigray, Ethiopia: a call to action. *The Lancet Global health* 2022;10(4):e469-e70. doi: https://dx.doi.org/10.1016/S2214-109X(22)00023-7
23. Teka H, Yemane A, Berhe E. War within a war: The challenges of providing gynecologic cancer care in the besieged Tigray region of northern Ethiopia. *Gynecol Oncol Rep* 2022;44:101093. doi: 10.1016/j.gore.2022.101093 [published Online First: 20221025]
24. Weldegerima AH, Tesfay H, Berhane S, et al. Tigray Siege and its impact on cardiology services in Mekelle University Hospital: a call to action. *European heart journal* 2022;43(33):3095-97. doi: 10.1093/eurheartj/ehac295
25. Berhe E, Ross W, Teka H, et al. Dialysis Service in the Embattled Tigray Region of Ethiopia: A Call to Action. *INTERNATIONAL JOURNAL OF NEPHROLOGY* 2022;2022 doi: 10.1155/2022/8141548

26. Favara M, Hittmeyer A, Porter C, et al. Young people, mental health, and civil conflict: Preliminary findings from Ethiopia's Tigray region. *Psychiatry Research Communications* 2022;2(1):100025. doi: https://doi.org/10.1016/j.psycom.2022.100025
27. Gebregziabher M, Amdeselassie F, Esayas R, et al. Geographical distribution of the health crisis of war in the Tigray region of Ethiopia. *BMJ global health* 2022;7(4) doi: https://dx.doi.org/10.1136/bmjgh-2022-008475
28. Hailu A, Gidey K, Zenebe D, et al. The Impact of the Northern Ethiopian Tigray War on Hypertensive Patients' Follow Up: a Brief Quantitative Study. *Pre-print* 2022 doi: 10.21203/rs.3.rs-2003722/v1
29. Weledegebriel MG, Abebe HT, Gidey K, et al. The Impact of War on HIV/AIDS Service Provision: in Rural Health Facilities of Tigray, Northern Ethiopia. *medRxiv* 2022:2022.11.29.22282873. doi: 10.1101/2022.11.29.22282873
30. Abay KA, Abay MH, Berhane G, et al. Access to health services, food, and water during an active conflict: Evidence from Ethiopia. *PLOS Global Public Health* 2022;2(11):e0001015. doi: 10.1371/journal.pgph.0001015
31. Phillips JF, Roy CM, Gebregziabher M. The international humanitarian response to famine in Tigray, Ethiopia:lessons from the Nigerian Civil War, 1967-1970. *Global health action* 2022;15(1):2107203. doi: https://dx.doi.org/10.1080/16549716.2022.2107203
32. Abebe H, Belaineh G. Key considerations: social science perspectives for emergency response to the conflict in Northern Ethiopia. Social Sceince in Humanitarian Action Platform, 2022.
33. Gesesew H, Berhane K, Siraj ES, et al. The impact of war on the health system of the Tigray region in Ethiopia: an assessment. *BMJ global health* 2021;6(11) doi: https://dx.doi.org/10.1136/bmjgh-2021-007328
34. HRW. "I Always Remember That Day": Access to Services for Survivors of Gender-Based Violence in Ethiopia's Tigray Region. Washington, USA: Human Rights Watch (HRW), 2021.
35. TRHB. Tigray health sector. Annual Bulletin 2021. Mekelle, Tigray: Tigray Regional Health Bureau, 2022.
36. WFP. Emergency Food Security Assessment: Tigray Region, Ethiopia. Geneva: World Food Program, 2022.
37. Hiluf K, Mustofa M, Fekade S, et al. Letter from Ayder Specialized hospital to Ethiopian Federal Minstry of Health: Tigray: a region where cancer diagnosis has become a death sentence In: Oncology, ed. Mekelle, Tigray, 2022:1-2.
38. TRHB. Letter from Tigray Regional Health Bureau (TRHB) to GAVI (Global Alliance Directorate Office): Ethiopia's Tigray ongoing war and Siege Mekelle, Tigray: TRHB, 2022.
39. Hiluf K. Letter from Ayder Specialized hospital to Ethiopian Diabetes Association: Request for life saving diabetes health care supplies. Mekelle, Tigray, 2022.
40. Berhe E, Kidu M, Teka H. Ethiopia's Tigray War: the agony of survival in kidney transplant recipients. *Journal of Nephrology* 2022;35(7):1797-99. doi: 10.1007/s40620-022-01380-3
41. Gebrearegay H, Berhe E, Lema HH, et al. Improvised, emergency peritoneal dialysis in children with acute kidney injury amid war in Tigray, Northern Ethiopia: two teaching cases. *Journal of Nephrology* 2022 doi: 10.1007/s40620-022-01386-x

List of excluded studies and reasons of exclusion

Wrong setting

1. Standley CJ, Jumaan AO, Sorrell EM, El Bcheraoui C. Editorial: Humanitarian Health in Conflict and Violence Settings. *FRONTIERS IN PUBLIC HEALTH* 2022; **10**.
2. Asrade G, Alemu K, Gebeye E, et al. The Burden of Internal Conflict on Expanded Programs on Immunization in Northwest Ethiopia: Implementation Science Study. *Ethiopian Journal of Health Development* 2021; **35**(3): 39-48.
3. Dadi AF, Mersha TB. WHO's surveillance system for attacks on health care is failing Ethiopia. *The Lancet* 2022; **399**(10331): 1225-6.
4. Dadi AF. The mental health consequences of war in northern Ethiopia: why we should be concerned. *The lancet Psychiatry* 2022; **9**(3): 194-5.
5. Bagcchi S. Ethiopia faces a measles outbreak amid a security crisis. *The Lancet Infectious diseases* 2022; **22**(5): 595.

Duplicated

6. Dadi A, Mersha TB. WHO's surveillance system for attacks on health care is failing Ethiopia (vol 399, pg 1225, 2022). *Lancet* 2022; **399**(10335): 1606-.
7. Gesesew H, Berhane K, Siraj ES. Erratum: The impact of war on the health system of the Tigray region in Ethiopia: an assessment (BMJ Global Health (2021) 6 (e007328) DOI: 10.1136/bmjgh-2021-007328). *BMJ Global Health* 2022; **7**(3).

8. Favara M, Hittmeyer A, Porter C, Singhal S, Woldehanna T. Young people, mental health, and civil conflict: Preliminary findings from Ethiopia's Tigray region. *Psychiatry Research Communications* 2022; **2**(1): 100025.
9. Masebo TM, Fikadu BK, Mehari E, et al. The need for rigour and balance in reporting the health impacts of conflict in Tigray, Ethiopia. *BMJ Global Health* 2022; **7**(3).
10. Gesesew H, Berhane K, Siraj ES, et al. The impact of war on the health system of the Tigray region in Ethiopia: an assessment. *BMJ global health* 2021; **6**(11).
11. Gesesew H, Berhane K, Siraj ES, et al. The impact of war on the health system of the Tigray region in Ethiopia: a response to complaints. *BMJ global health* 2022; **7**(3).

Wrong outcomes

12. Akresh R, Caruso GD, Thirumurthy H. Detailed geographic information, conflict exposure, and health impacts. *WORLD DEVELOPMENT* 2022; **155**.
13. Fisher J. #HandsoffEthiopia: 'Partiality', Polarization and Ethiopia's Tigray Conflict. *GLOBAL RESPONSIBILITY TO PROTECT* 2022; **14**(1): 28-32.
14. Piombo J, Englebert P. The war on terror in context: domestic dimensions of Ethiopia and Kenya's policies towards Somalia. *THIRD WORLD QUARTERLY* 2022; **43**(5): 1176-96.
